# The Possible Role of Metformin and Fibroblast Growth Factor‐21 in Multiple Sclerosis Neuropathology: Birds of a Feather Flock Together

**DOI:** 10.1111/ejn.70067

**Published:** 2025-04-02

**Authors:** Ahmad A. Abulaban, Hayder M. Al‐kuraishy, Ali I. Al‐Gareeb, Eman A. Ahmed, Mubarak Alruwaili, Athanasios Alexiou, Marios Papadakis, Gaber El‐Saber Batiha

**Affiliations:** ^1^ College of Medicine King Saud bin Abdulaziz University for Health Sciences Riyadh Saudi Arabia; ^2^ Division of Neurology, King Abdulaziz Medical City Ministry of the National Guard Health Affairs Riyadh Saudi Arabia; ^3^ King Abdullah International Medical Research Center Riyadh Saudi Arabia; ^4^ Department of Clinical Pharmacology and Medicine, College of Medicine Al‐Mustansiriya University Bagdad Iraq; ^5^ Department of Pharmacology, Faculty of Veterinary Medicine Suez Canal University Ismailia Egypt; ^6^ Department of Internal Medicine, College of Medicine Jouf University Sakaka Saudi Arabia; ^7^ University Centre for Research & Development Chandigarh University Mohali Punjab India; ^8^ Department of Research and Development Funogen Athens Greece; ^9^ Department of Surgery II University Hospital Witten‐Herdecke, University of Witten‐Herdecke Wuppertal Germany; ^10^ Department of Pharmacology and Therapeutics, Faculty of Veterinary Medicine Damanhour University Damanhour Egypt

**Keywords:** CNS plaques, demyelination, metformin, multiple sclerosis, oxidative stress, remyelination

## Abstract

Multiple sclerosis (MS) is a progressive demyelinating disease of the CNS, characterized by inflammation, the formation of CNS plaques, and damage to the neuronal myelin sheath (Graphical abstract). Fibroblast growth factor 21 (FGF21) is involved in various metabolic disorders and neurodegenerative diseases. FGF21 and its co‐receptor β‐Kloth are essential in the remyelination process of MS. Metformin, an insulin‐sensitizing drug that is the first‐line treatment for type 2 diabetes mellitus (T2DM), may have a potential neuroprotective impact by up‐regulating the production of FGF21, which may prevent the onset of neurodegenerative diseases including MS. The purpose of this review is to clarify how metformin affects MS neuropathology mechanistically via modifying FGF21. Metformin increases the expression of FGF21. Metformin also increases the expression of β‐Klotho, modulates oxidative stress, reduces glutamate‐induced excitotoxicity, and regulates platelet function and coagulation cascades. In conclusion, metformin can enhance the functional activity of FGF21 in counteracting the development and progression of MS. Preclinical and clinical studies are warranted in this regard.

## Graphical abstract

Predisposing factors of multiple sclerosis and Role of Metformin and Fibroblast Growth Factor‐21: A key predisposing factor for multiple sclerosis (MS) is genetic susceptibility, which is associated with an increased risk of the disease. Environmental factors, such as low vitamin D levels, smoking, and viral infections, also contribute to the developing MS. The figure highlights the potential molecular mechanisms and pathways through which metformin and FGF21 could modulate MS disease progression, providing insights into novel therapeutic approaches.

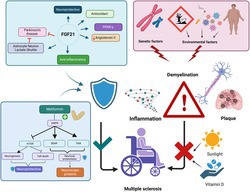

**Abbreviations**
ad
: Alzheimer's disease
AngII: angiotensin II
Aβ: amyloid beta
BDNF: brain‐derived neurotrophic factor
COX2: cyclooxygenase 2
EBV: Epstein–Barr virus
FGF21: fibroblast growth factor 21
GFR: glomerular filtration rate
GLP‐1: glucagon‐like peptide 1
GLUT4: glucose transporter type 4
HLA: human leukocyte antigen
HMGCS2: 3‐hydroxy‐3‐methylglutaryl‐CoA synthase
iNOS: nitric oxide synthase
LRRK2: leucine‐rich repeat kinase 2
MATE1: multidrug and toxin extrusion 1
MHCII: major histocompatibility complex class II
MMP9: matrix metalloproteinase 9
MS: multiple sclerosis
OCT2: organic cation transporter 2
OPCs: oligodendrocyte precursor cells
PD: Parkinson's disease
PI3K: phosphoinositol 3 kinase
PIK1: Parkinson's disease kinase 1
PMAT: plasma membrane monoamine transporter
PPAR‐α: peroxisome proliferator‐activated receptor alpha
PPAR‐γ: PPAR gamma
SIRT1: Sirtuin‐1
T2DM: type 2 diabetes mellitus
tPA‐1: plasminogen activator inhibitor
TrkB: tropomyosin receptor tyrosine kinase B
VEGF: vascular endothelial growth factor
βTG: β thromboglobulin

## 1 Introduction

Multiple sclerosis (MS) is a demyelinating illness of the central nervous system (CNS) (Dobson and Giovannoni 2019). MS is the most common inflammatory disease that impairs motor and sensory neural signal transmission(Dobson and Giovannoni 2019; Kumar et al. 2011; Vecchio et al. 2017). MS is presented clinically by unilateral visual loss, double vision, muscular weakness, and motor‐sensory incoordination (Kumar et al. 2011; Vecchio et al. 2017). MS affects 2.8 million subjects globally, with prevalence varying among different populations (Dutta and Trapp 2014; Buscarinu et al. 2019; Lane et al. 2022). In 2022, about one million people in the United States were living with MS (McGinley et al. 2021). The disease is more common in women, typically affecting individuals between the ages of 20 and 50 (Zeydan and Kantarci 2020).

The central pathophysiology of MS is failure of myelin production or damage to the myelin sheath by immune cells (Buscarinu et al. 2019). In genetically susceptible individuals, abnormal immune responses to certain environmental factors can trigger cell‐mediated, leading to axonal demyelination (Derada Troletti et al. 2019).CNS plaque development, which is a hallmark of MS neuropathology, is characterized by inflammation and damage to the neuronal myelin sheath (Derada Troletti et al. 2019). Sunlight exposure and adequate levels of vitamin D have been found to protect against the development of MS (Derada Troletti et al. 2019).

Multiple focal patches of demyelination scattered across the cerebral cortex, deep gray matter, spinal cords, and white matter of the brain are characteristics of these plaques (Al‐Kuraishy, Al‐Gareeb, et al. 2024; Al‐Kuraishy, Jabir, et al. 2024; Al‐Kuraishy et al. 2023a; Al‐Kuraishy, Sulaiman, et al. 2024; Alruwaili et al. 2023; Khatir et al. 2020; Lassmann et al. 2001). In MS, oligodendrocytes, which are responsible for producing the myelin sheath, are principally impacted (Lan et al. 2017; Sedel et al. 2016). Partial remyelination can occur during the remission phase, while demyelination often recurs during the relapse stage, leading to plaque formation at multiple sites within the CNS (Ponath et al. 2018). MS is considered as an immune‐mediated disease involving both the humoral and cellular arms of the immune system (Martino et al. 2002). Auto‐reactive T lymphocytes in the peripheral system initiate inflammatory alterations in MS (Liu et al. 2007). It has been shown that molecular mimicry triggers polyclonal activation of peripheral auto‐reactive T cells (Elsayed et al. 2022). Auto‐reactive T cells have the ability to cross through the blood–brain barrier (BBB) via attaching to VCAM‐1 on endothelial cells and integrins on immune cells (Rice et al. 2005). Inflammation and pro‐inflammatory cytokines (Gerdes et al. 2020; Hedström et al. 2020; Aloisi and Cross 2022; Coles 2008; Marrie 2004; Sf 2021) lead to an up‐regulation of VCAM‐1 and integrin expression (Dyment et al. 2004). In addition, T cells stimulate the production and secretion of matrix metalloproteinase (MMPs), which promote the entry of T cells and contribute to the myelin degeneration (Mohammadhosayni et al. 2020; Martin et al. 2021; Balasa et al. 2020; James et al. 2020).

**FIGURE 1:**
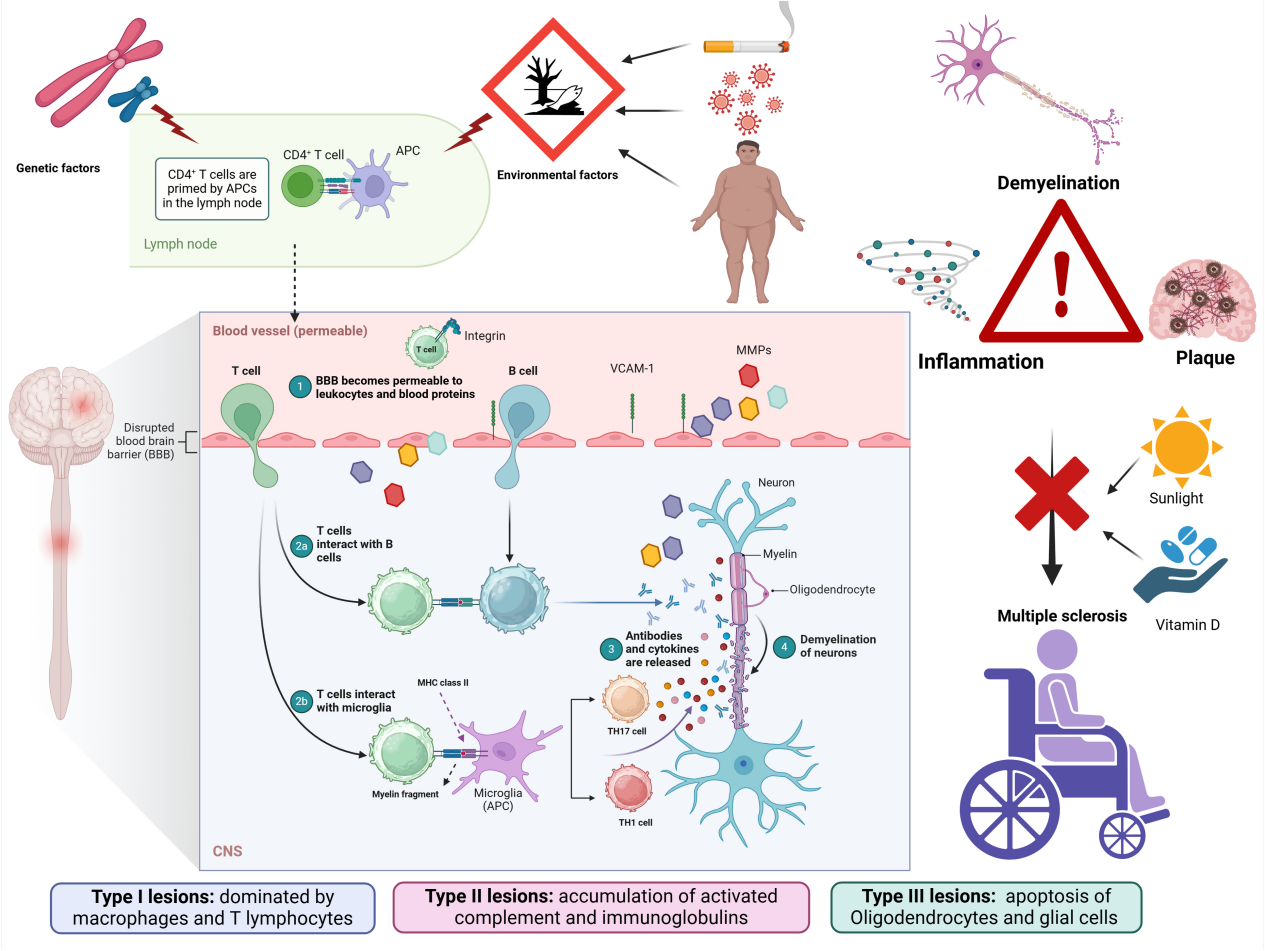
Pathophysiology of MS.

These neuropathological changes induce axonal damage and injury of the myelin sheath (Figure 1).

Acute attacks of MS are treated with high doses of corticosteroids such as methylprednisolone either orally or intravenously (Hauser and Cree 2020; Pitt et al. 2022; Makhani and Tremlett 2021; Granziera et al. 2021; Sakakibara 2019; Mayo et al. 2019; Alhossan et al. 2022; Teoli et al. 2021). Plasmapheresis is recommended when corticosteroid therapy is ineffective. Disease‐modifying therapies for chronic MS management include mitoxantrone, glatiramer, and interferons (Tintore et al. 2019). Additionally, about 50% of MS patients use alternative and complementary medicine, including biotin, vitamin D, and cannabis (Vespignani 2020). Earlier studies have demonstrated that metformin can attenuate the inflammatory reactions in MS (Xu et al. 2007; Zhou et al. 2012). Moreover, fibroblast growth factor 21 (FGF21), a growth factor involved in various metabolic disorders and neurodegenerative diseases (Kliewer and Mangelsdorf 2010), is affected by metformin (M. Zhang et al. 2013). Conferring to these findings, this review aims to discuss the immunoinflammatory effect of metformin in MS in relation to the expression of FGF21.

## 2 The Physiological Role of FGF21: An Overview

FGF21 is a peptide hormone synthesized by hepatocytes (Al‐Kuraishy et al. 2023b). Along with FGF19 and FGF21, FGF23 forms part of the FGF family, which is involved in regulating angiogenesis, cell mitosis, and differentiation (Al‐kuraishy et al. 2023c). FGF21 is highly expressed in the liver, adipose tissues, and pancreas (Nygaard et al. 2014). It is also produced by skeletal muscles and other tissues, with its expression enhanced by phosphoinositol 3 kinase (PI3K) (Sun et al. 2021). The expression of FGF21 is influenced by various cellular signaling; for example, the liver X receptor (LXR) inhibits the expression of FGF21 (Pérez‐Martí et al. 2017; Rodgers et al. 2019; Uebanso et al. 2012). FGF21 improves glucose, lipid metabolism and energy expenditure (Pérez‐Martí et al. 2017). FGF21 binds four types of FGF receptors (FGFR1–4), and its interaction with these receptors is enhanced by β‐Klotho, a transmembrane protein that serves as a co‐receptor for FGF21 (Min et al. 2018). The expression of FGF21 varies under different pathophysiological conditions, for example, exercise and fasting increase muscular and hepatic FGF21 expression, correspondingly (Cuevas‐Ramos et al. 2010; K. Li et al. 2012). Interestingly, low‐protein diet enhances FGF21 expression, thereby improving the metabolic profile (Larson et al. 2019). In addition, peroxisome proliferator‐activated receptor alpha (PPAR‐α) induces the expression of FGF21 in the liver, whereas PPAR gamma (PPAR‐γ) induces the expression of FGF21 in adipose tissue (Yu 2015). Moreover, FGF21 expression is induced by sirtuin‐1 (SIRT1) and mitochondrial 3‐hydroxy‐3‐methylglutaryl‐CoA synthase (HMGCS2) (Li et al. 2014).

**FIGURE 2:**
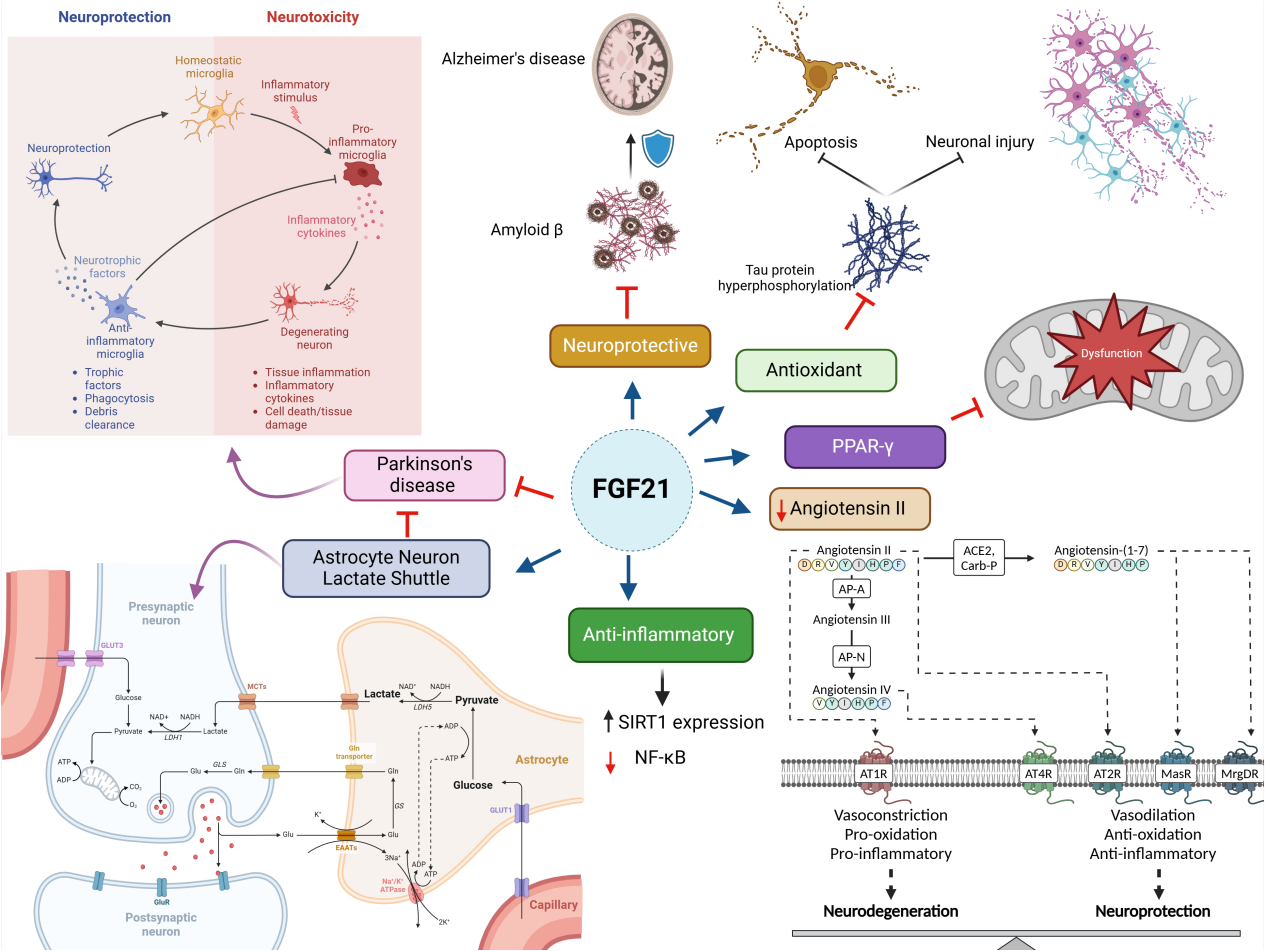
Neuroprotective effects of FGF21.

It has been shown that FGF21 exhibits neuroprotective effects against numerous neurodegenerative diseases. For instance, it reduces the neurotoxicity caused by amyloid beta (Aβ) in Alzheimer's disease (AD) (Chen et al. 2019). In vitro and in vivo studies have confirmed that FGF21 inhibits tau protein hyperphosphorylation‐induced neuronal injury and apoptosis through its antioxidant effects (Chen et al. 2019). Moreover, FGF21 has neuroprotective effects against Parkinson's disease (PD) by promoting the anti‐inflammatory phenotype (Yang et al. 2021). This anti‐inflammatory effect of FGF21 is primarily mediated by increasing the expression of SIRT1 and inhibiting of inflammatory signaling pathways such as NF‐κB (Yang et al. 2021). Sun et al. (2020) found that FGF21 modulates the neuronal‐astrocyte lactate shuttle pathway, which is dysregulated in AD. FGF21 also attenuates neuroinflammation and promotes neurogenesis in various neurodegenerative diseases including AD and PD (Woodbury and Ikezu 2014). Furthermore, FGF21 inhibits mitochondrial dysfunction by up‐regulating the production of peroxisome proliferator‐activated‐γ coactivator 1 α (PGC‐1α) (Restelli et al. 2018; Mäkelä et al. 2014). Furthermore, FGF21 reduces angiotensin II (AngII)‐induced cerebrovascular aging and related oxidative stress and inflammatory conditions (X.‐M. Wang et al. 2016). These verdicts indicated that FGF21 significantly protects neurons from the development and progression of neurodegenerative disorders (Figure 2).

## 3 Role of FGF21 in MS

FGF21 is an important growth factor that promotes the differentiation and proliferation of oligodendrocyte precursor cells, which is crucial for the remyelination process (Kurosu et al. 2007; Adams et al. 2012). Of note, FGF21 and its co‐receptor β‐Kloth are essential for remyelination in MS (Kuroda et al. 2017). It has been reported that the CSF level of FGF21 is approximately 40% of its level in circulation in healthy subjects (Tan et al. 2011) due to limited entry of FGF21 into the CNS through the BBB, suggesting that peripheral administration of FGF21 may not effectively effective in the management of MS. However, disruption of BBB integrity in neurodegenerative diseases facilitates the entry of FGF21 into CNS (Chen et al. 2018). Nonetheless, peripherally administration FGF21 has been proven to be effective in promoting remyelination in MS (Kuroda et al. 2017). Disruption of BBB is a critical factor in the pathogenesis of MS as it prevents the entry of immune and inflammatory cells to the CNS (Ortiz et al. 2014). Interestingly, FGFR1 and FGFR1 are prominently expressed in oligodendrocyte precursor cells, suggesting a significant role in the remyelination process (Fortin et al. 2005). In addition, FGF21 protects neurons from glutamate‐induced excitotoxicity, which involved in the pathogenesis of MS via inhibition of oxidative and inflammatory reactions (Linares et al. 2020; Xu et al. 2021). As well, FGF21 attenuates AD‐induced neuroinflammation by inhibiting NF‐κB, TLR4, and heat shock protein 90 (Amiri et al. 2018). Neuroinflammation is closely linked with progressive neurodegeneration and demyelination in MS (Koudriavtseva and Mainero 2016). Therefore, FGF21 can attenuate MS neuropathology by targeting both glutamate‐induced excitotoxicity and neuroinflammation. A case–control study involving 50 MS patients and 33 healthy controls revealed increased serum levels of FGF21 and vascular endothelial growth factor (VEGF) in MS patients compared to healthy controls (Su et al. 2006). These findings underscore the potential significance of FGF21 and VEGF in the pathophysiology of MS.

In MS, the distribution and progression of sclerotic lesions in the brain stem and spinal cord can compromise the integrity of the autonomic nervous system, leading to severe autonomic dysfunction (Autonomic 2003). Research has indicated that FGF21 improves sympathetic drive and enhances autonomic function in mice (Douris et al. 2015). FGF21 plays a versatile role in regulating energy expenditure by activating the sympathetic nervous system (BonDurant and Potthoff 2018). Furthermore, FGF21 improves cognitive function by promoting hippocampal neurogenesis in mouse models with traumatic brain injury (Shahror et al. 2020). Yu et al. (2015) revealed that FGF21 attenuates D‐galactose‐induced aging in mice by boosting hippocampal neurogenesis and preventing of oxidative stress. FGF21 also improves synaptic plasticity, thereby improving memory and learning capabilities (Reuss and Bohlen und Halbach 2003). In the context of MS, auto‐reactive T lymphocytes trigger inflammatory changes in the MS (Singhal et al. 2016), and inhibition of these cells could mitigate MS neuropathology. FGF21 has the remarkable ability to suppress the activity and growth of T lymphocytes in mice. This suppression is accompanied by a decrease in the production of pro‐inflammatory cytokines (Singhal et al. 2016). FGF21 can suppress NF‐κB, which is a key regulator of B and T lymphocytes (Yu et al. 2016). Therefore, FGF21 plays a crucial role in regulating immunoinflammatory response in MS neuropathology.

FGF21 binds to FGF receptors, a process that enhanced by β‐Klotho, a transmembrane protein that acts as a co‐receptor for FGF21 (Yu 2015). This co‐receptor exerts differential neuroprotective effects against neurodegenerative diseases, including MS. β‐Klotho agonists mimic the action of FGF21 (Min et al. 2018), suggesting a potential role of this receptor in MS. A study conducted by Aleagha et al. (2015) showed that CSF level of β‐Klotho was reduced in MS patients compared to controls. β‐Klotho promotes the maturation of oligodendrocyte precursor cells and improves myelination in MS patients (Scazzone et al. 2019). Similarly, a case–control study revealed that β‐Klotho mRNA expression in the peripheral blood mononuclear cells was reduced in MS patients compared to controls (Karami et al. 2017). Notably, Klotho protein improves and maintains vitamin D metabolism, which acts as a neurosteroid mitigating different neurodegenerative diseases, mainly MS (Dërmaku‐Sopjania et al. 2021). Vitamin D deficiency is regarded as a risk factor for the development and progression of MS (Miclea et al. 2020). Therefore, Klotho protein and FGF21 regulate different inflammatory and metabolic pathways implicated in MS neuropathology.

## 4 The Neuroprotective Effects of Metformin

**FIGURE 3:**
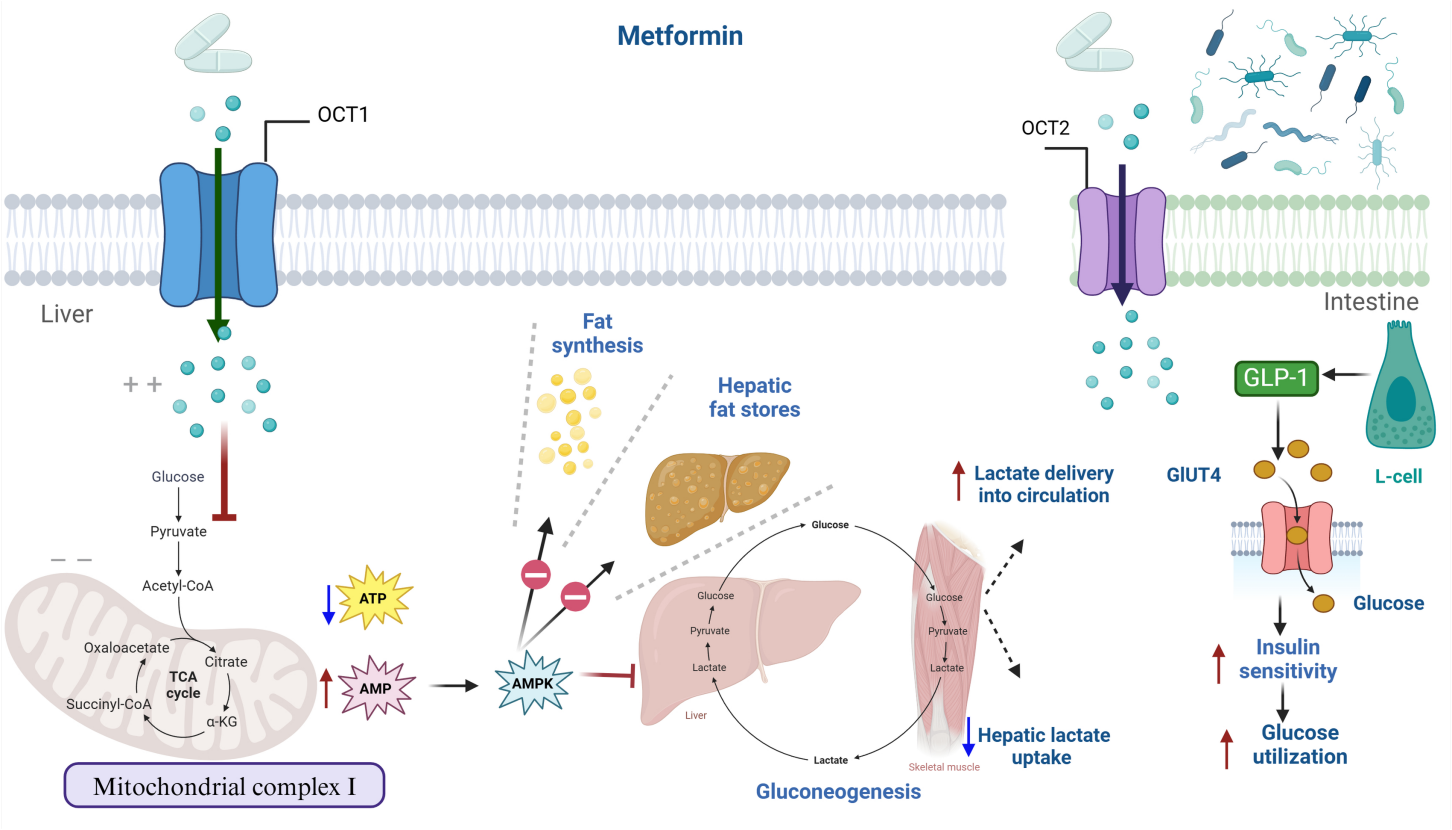
Pharmacology of metformin.

Metformin is an insulin‐sensitizing drug belonging to the biguanide class; it is used as first‐line therapy in the management of T2DM due to its ability to decrease peripheral insulin resistance (IR) (Al‐Kuraishy, Sami, et al. 2020; Al‐Kuraishy, Al‐Gareeb, Saad, and Batiha 2023). Metformin is an orally administered medication, absorbed in the small intestine through the highly expressed plasma membrane monoamine transporter (PMAT) in enterocytes (Al‐Kuraishy, Al‐Gareeb, Alblihed, Cruz‐Martins, et al. 2021; Al‐Kuraishy, Al‐Gareeb, Waheed, and Al‐Maiahy 2018; Al‐Kuraishy et al. 2019; Al‐Kuraishy et al. 2016; Al‐Kuraishy, Al‐Gareeb, Alexiou, et al. 2022; He 2020). By inhibiting mitochondrial complex I, metformin prevents the synthesis of ATP, which raises adenosine monophosphate protein kinase levels and increases the AMP:ATP ratio (AMPK). AMPK enhances anaerobic glucose metabolism and insulin sensitivity in enterocytes (Abdul‐Hadi, Naji, Shams, et al. 2020) (Figure 3). It also encourages the gut microbiota to utilize glucose by stimulating the release of glucagon‐like peptide 1 (GLP‐1) from intestinal L cells (Al‐Kuraishy, Al‐Gareeb, Albogami, et al. 2022). Moreover, it has pleiotropic properties such as antioxidant and anti‐inflammatory effects (Al‐Kuraishy, Al‐Gareeb, Alblihed, Guerreiro, et al. 2021; Al‐Kuraishy, Al‐Gareeb, Albogami, et al. 2022; Foretz et al. 2019; Al‐Kuraishy and Al‐Gareeb 2016; Rasheed et al. 2019; Al‐Nami et al. 2020; Al‐kuraishy et al. 2016; Flory and Lipska 2019; Abdul‐Hadi, Naji, Shams, Sami, et al. 2020; Al‐Kuraishy, Al‐Gareeb, Alsayegh, et al. 2023; Trueck et al. 2019; LaMoia and Shulman 2021; Tarry‐Adkins et al. 2021).

Metformin has potential neuroprotective effects against numerous neurodegenerative diseases such as AD and PD. Mounting evidence suggests that metformin decreases the risk of AD in T2DM patients (Nath et al. 2009; Herath et al. 2016). Compared with other diabetic treatments, metformin has been reported to enhance working memory and executive brain functions in T2DM patients (Luchsinger et al. 2016; Imfeld et al. 2012). It has been shown that prolonged metformin treatment > 4 years reduces the risk of AD, PD, and Huntington's disease (Herath et al. 2016). Conversely, numerous studies showed an increased risk of cognitive impairment and AD with long‐term metformin therapy (Imfeld et al. 2012; Moore et al. 2013). The neuroprotective effect of metformin is primarily mediated through AMPK, which mitigates the aggregation of Aβ and hyperphosphorylation of tau proteins (J. Li et al. 2012). In addition, metformin enhances neurogenesis, angiogenesis, and synaptic plasticity and induces autophagy (Venna et al. 2014). Metformin improves the levels of brain‐derived neurotrophic factor (BDNF), which inhibit neuroinflammation and protect neurons from oxidative stress (Chen et al. 2016). C. Wang et al. (2016) showed that metformin exerted a neuroprotective effect via inhibiting apoptosis and activating autophagy in mice with experimental spinal cord injury. It has been reported that chronic AMPK activation by metformin has protective effects against ischemic stroke. However, acute metformin treatment exacerbates acute ischemic stroke (Turkistani et al. 2024; Mima et al. 2016; Sharma et al. 2021). Consequently, timing of metformin administration and its duration are crucial factors in mitigating acute ischemic stroke. Notably, in T2DM patients, metformin usage lowers the risk and severity of ischemic stroke (Mima et al. 2016). A prospective study demonstrated that treatment with metformin before the onset of acute ischemic stroke decreases the disease severity in T2DM patients (Mima et al. 2016). In recent years, metformin has been repurposed for the management of age‐related neurodegenerative disorders and ischemic stroke via the modulation of AMPK and different signaling pathways (Sharma et al. 2021).

**FIGURE 4:**
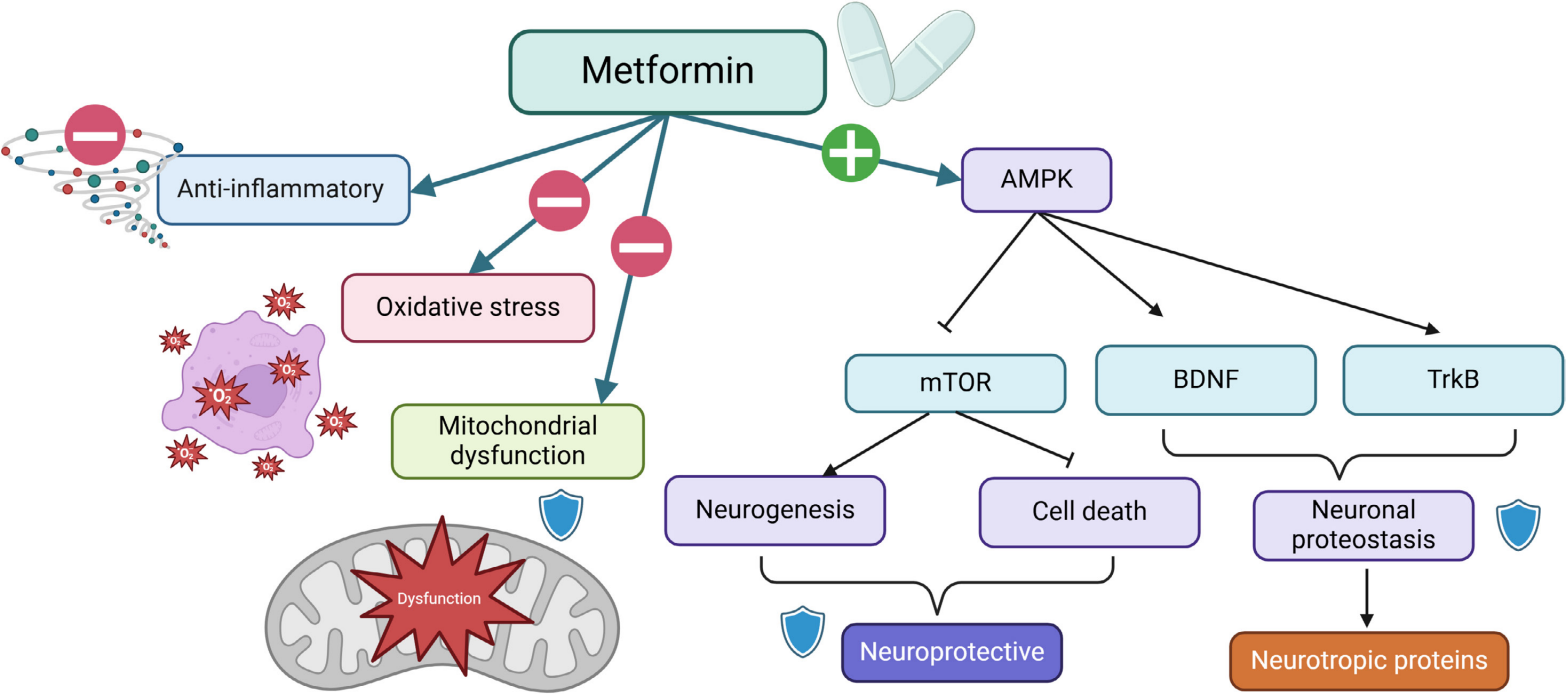
Anti‐epileptic effect of metformin.

Additionally, metformin has beneficial effects against the development of epilepsy and exhibits anti‐epileptic properties by ameliorating oxidative stress, inhibiting mTOR‐induced neuroinflammation, and modulating of neuronal AMPK signaling (Alnaaim et al. 2023). Metformin attenuates abnormal neurogenesis, neuronal cell deaths, and neuroinflammation in epilepsy (Sanz et al. 2021). AMPK improves neuronal proteostasis by modulating BDNF and tropomyosin receptor tyrosine kinase B (TrkB), which play roles in epileptogenesis (Sanz et al. 2021). Singh et al. emphasized that metformin is a promising anti‐epileptic agent by modulating epileptogenesis process via inhibiting oxidative stress, mTOR, and inflammatory signaling pathways (Singh et al. 2022). An experimental study established the efficacy of metformin in attenuating pilocarpine‐induced seizure in rats (Mehrabi et al. 2018). These findings propose that metformin may exert anti‐epileptic effects through modulation of TrkB, BDNF, oxidative stress, and inflammatory signaling pathways. Furthermore, metformin boosts the expression of the neuroprotective protein progranulin, which helps to reduce neuronal excitability and prevent seizure development in temporal lobe epilepsy (Alrouji et al. 2024; Paudel et al. 2020; Vazifehkhah et al. 2020). Thus, metformin exhibits anti‐epileptic effects by regulating astrogliosis and the release of progranulin (Figure 4).

Certainly, *PARKIN* is recognized as a gene that protects against mitochondrial damage in PD and other neurodegenerative brain disorders (Mehrabi et al. 2018). The neuroprotective benefits of metformin are attributed to its enhancement of *PARKIN* gene expression (Mehrabi et al. 2018). Furthermore, metformin modulates several genes implicated in neurodegenerative brain diseases such as leucine‐rich repeat kinase 2 (*LRRK2*) and PD‐like Parkinson's disease kinase 1 (*PIK1*) genes (Alrouji et al. 2024; Paudel et al. 2020).

Overall, metformin offers a wide range of neuroprotective benefits by promoting neurogenesis, regulating cognitive dysfunction, counteracting aging processes, mitigating mitochondrial dysfunction and oxidative stress, enhancing autophagy, and modulating the expression of neuroprotective genes.

## 5 Role of Metformin in MS

It has been reported that metformin attenuates autoimmune response in the CNS in an animal model of MS. Metformin is effective against experimental autoimmune encephalomyelitis (EAE) via the inhibition of T cell‐mediated inflammation through AMPK‐dependent pathway in mice (Nath et al. 2009). Metformin attenuates the induction of EAE by restricting the infiltration of mononuclear cells into the CNS, down‐regulating the expression of pro‐inflammatory cytokines, cell adhesion molecules, matrix metalloproteinase 9, and chemokine (Onohuean et al. 2021; El‐Saber Batiha et al. 2022; Al‐Kuraishy, Al‐Gareeb, Alsayegh, et al. 2023; Hasan Khudhair et al. 2022; Al‐Kuraishy, Al‐Gareeb, Alqarni, et al. 2021; Al‐Kuraishy, Al‐Gareeb, Waheed, and Al‐Maiahy 2018). Moreover, the AMPK activity and lipids alterations (total phospholipids and in free fatty acids) are restored by metformin treatment in the CNS of treated EAE animals, suggesting the possible involvement of AMPK. Metformin activated AMPK in macrophages and thereby inhibited biosynthesis of phospholipids as well as neutral lipids and also down‐regulated the expression of endotoxin‐induced pro‐inflammatory cytokines and their mediators such as iNOS and cyclooxygenase 2. It also attenuated IFN‐gamma and IL‐17‐induced iNOS and cyclooxygenase 2 expression in RAW267.4 cells, further supporting its anti‐inflammatory property. Metformin inhibited T cell‐mediated immune responses including Ag‐specific recall responses and production of Th1 or Th17 cytokines, while it induced the generation of IL‐10 in spleen cells of treated EAE animals (Nath et al. 2009). It has been revealed that metformin via AMPK can inhibit T cell‐mediated autoimmunity in EAE (Paintlia et al. 2013). The expressions of neurotrophic factors and of signatory genes of oligodendrocytes lineages were increased in the CNS of metformin‐treated EAE animals. Similarly, metformin protects oligodendrocytes via AMPK activation in mixed glial cultures. Therefore, AMPK activators, including metformin, have the potential to limit neurologic deficits in MS and related neurodegenerative disorders. Metformin can limit the infiltration of immunoinflammatory cells into the CNS and inhibit the expression of pro‐inflammatory cytokines, matrix metalloproteinase 9 (MMP9), chemokines, and adhesion molecules (Sag et al. 2008; Alsubaie et al. 2022; Al‐Kuraishy, Al‐Gareeb, Al‐Niemi, et al. 2022; Moubarak et al. 2021; Al‐Kuraishy and Al‐Gareeb 2016; Babalghith et al. 2022; Al‐Kuraishy, Al‐Gareeb, and Batiha 2022; Al‐Kuraishy, Al‐Naimi, et al. 2020; Rasheed et al. 2019). Furthermore, metformin blocks the expression of inducible nitric oxide synthase (iNOS) and cyclooxygenase 2 via inhibiting the IL‐17 and INF‐γ signaling pathway, as well as T cell‐mediated Th1 cytokines (Sag et al. 2008). These findings indicated that metformin in virtue of its anti‐inflammatory could be effective against MS neuropathology, as documented recently (Dziedzic et al. 2020).

Metformin has a potent remyelinating effect by enhancing the activity and differentiation of oligodendrocyte precursor cells (OPCs) in rats (Neumann et al. 2019; Alorabi et al. 2022; Al‐Kuraishy, Al‐Gareeb, Qusti, et al. 2021; Alkuraishy, Al‐Gareeb, and Waheed 2018; Batiha et al. 2021; Al‐Kuraishy, Al‐Gareeb, Abdullah, et al. 2021; Al‐Kuraishy, Al‐Gareeb, Qusty, et al. 2022). Of note, the differentiation potential of adult rodent OPCs decreases with age. Aged OPCs become unresponsive to pro‐differentiation signals, signifying intrinsic constraints on therapeutic approaches aimed at enhancing OPC differentiation. This decline in functional capacity is associated with hallmarks of cellular aging, including decreased metabolic function and increased DNA damage. Fasting or treatment with metformin can reverse these changes and restore the regenerative capacity of aged OPCs, improving remyelination in aged animals following focal demyelination. Aged OPCs treated with metformin regain responsiveness to pro‐differentiation signals, suggesting synergistic effects of rejuvenation and pro‐differentiation therapies (Neumann et al. 2019). These findings provide insight into aging‐associated remyelination failure and suggest therapeutic interventions for backing such declines in chronic disease. Furthermore, metformin can inhibit gliosis in MS by reducing the functional activity of microglia and astrocytes, thereby enhancing oligodendrocytes' function and myelination process (Largani et al. 2019; Al‐Kuraishy, Al‐Gareeb, et al. 2020; Al‐Kuraishy, Hussien, et al. 2020; Al‐Kuraishy, Al‐Maiahy, et al. 2020; Batiha et al. 2023; Al‐Thomali et al. 2022; Mostafa‐Hedeab et al. 2022; Hussien et al. 2018; Al‐Kuraishy, Al‐Gareeb, Saad, and Batiha 2023; Al‐Kuraishy, Al‐Gareeb, Elekhnawy, and Batiha 2022). Furthermore, it has been suggested that metformin exerts its beneficial influence through AMPK pathway. Metformin up‐regulates the expression of mitochondrial biogenesis genes and ameliorates the oxidative stress in the cuprizone‐induced demyelination. As well, metformin reduces astrogliosis and microgliosis via AMPK signaling (Largani et al. 2019). Consistently, Largani et al. (2019) illustrated that metformin has a protective effect on the oligodendrocytes by reducing oxidative stress and mitochondrial dysfunction in the cuprizone‐induced MS model in mice. Metformin also enhances myelin recovery and mitigates behavioral deficits in animal models of MS by modulating oligodendrogenesis via an AMPK‐dependent pathway (Sanadgol et al. 2020).

On the other hand, metformin exerts a neuroprotective effect against MS pathogenesis through AMPK‐independent mechanism. Metformin is not only a great regenerating and remyelinating agent to activate endogenous neural precursors but also a promising candidate as a preconditioning reagent to maximize the grafting and differentiation potential of transplanted exogenous neural stem cells in vivo. It has been shown that pre‐treatment of human induced pluripotent stem cell‐derived neural stem cells with metformin before transplantation into the rat stroke brain can enhance their capability to graft and differentiate into neurons, astrocytes, and oligodendrocytes in vivo (Ould‐Brahim et al. 2018). Metformin has been shown to promote the proliferation and differentiation of neuroblasts, formation of oligodendrocytes and impact pericyte maturity and coverage following ischemic stroke. Interestingly, metformin's ability to promote neural regeneration following stroke may be sex‐dependent (Ruddy et al. 2019). Studies have confirmed the beneficial efficacy of metformin on fibrinolysis (Krysiak et al. 2013). Inhibitors of hemostasis proteases, such as plasminogen activator inhibitor 1 (PAI‐1), tissue factor inhibitor (TFPI), and thrombomodulin (TM), are the key regulators of fibrinolysis and coagulation. The PAI‐1 level has been reported to be higher in MS patients during exacerbations. The genetic polymorphisms of PAI‐1 are linked to lower PAI‐1 plasma levels and are associated with increased risk of developing MS syndrome (Lovrecic et al. 2008). Furthermore, there is a statistically positive correlation between expanded disability status scale (EDSS) scores and TM levels in MS patients (Balkuv et al. 2016). Studies conducted on 138 MS patients (85 RRMS and 53 P‐MS) have demonstrated higher PAI‐1 and TFPI levels in MS patients compared to healthy individuals. Studies have shown that metformin decreases coagulation factor VII and the circulating level of PAI‐1. Furthermore, it interferes with factor XIII, cross‐linking activity and fibrin polymerization (Standeven et al. 2002). Interestingly, metformin inhibits inflammation‐induced neurodegeneration and demyelination in MS by inhibiting the expression of NF‐κB and the expression of pro‐inflammatory cytokines (Sanadgol et al. 2020). Moreover, metformin increases the expression of PPARα (Maida et al. 2011), which is dysregulated in MS (Storer et al. 2005). Activation of PPARα has been shown to have beneficial effects in MS models. For instance, fenofibrate, a PPARα agonist, attenuates LPS‐induced expression and the release of pro‐inflammatory cytokines from activated astrocytes in EAE (Xu et al. 2007). Fenofibrate achieves this by inhibiting MyD88‐mediated TLR4 signaling and the expression of IL‐12 (Xu et al. 2007). Therefore, metformin, through the activation of PPARα, can potentially reduce MS neuropathology by modulating inflammatory pathways and reducing the pro‐inflammatory cytokine burden. This AMPK‐independent mechanism adds to the multifaceted approach of metformin in ameliorating MS‐related neuroinflammation and promoting neuroprotection.

Furthermore, metformin affects adipokines, including leptin and adiponectin (Dludla et al. 2021), which are involved in the pathogenesis of autoimmunity and MS (Harroud et al. 2021). Leptin promotes pro‐inflammatory signaling whereas adiponectin enhances anti‐inflammatory cytokines in MS (Nygaard et al. 2012). A case–control study included 25 MS patients and 25 healthy controls observed that plasma leptin and adiponectin were increased and decreased respectively in MS patients compared to controls (Nygaard et al. 2012). Metformin has been observed to increase adiponectin levels and reduce leptin levels (Dludla et al. 2021), thereby potentially modulating adipokines‐mediated MS neuropathology. A placebo‐controlled clinical trial investigating the possible role of metformin in MS neuropathology has been initiated (Li et al. 2020; Aatsinki et al. 2014), and we await the outcomes. A cohort study revealed that metformin use for 6–24 months in MS patients reduced brain sclerotic lesions by lowering levels of IL‐17 and INF‐γ, while augmenting AMPK activity and anti‐inflammatory regulatory T cells (Negrotto et al. 2016).

Moreover, imeglimin, which has a chemical structure close to metformin, is a novel oral antidiabetic drug modulating mitochondrial functions, has a neuroprotective effect against ischemia‐induced brain damage and neurological deficits (Zemgulyte et al. 2022). Findings from preclinical study observed that imeglimin by inhibiting of mitochondrial permeability transition pore (mPTP) and microglial activation attenuates ischemia‐induced brain damage in rats (Zemgulyte et al. 2022). In addition, imeglimin reduces LPS‐induced NLRP3 inflammasome activation by inhibiting mPTP opening in THP‐1 macrophages (Lee et al. 2024). NLRP3 inflammasome activation is linked with development and progression of MS (Olcum et al. 2020). Therefore, the structural similarity between metformin and imeglimin may explain the neuroprotective effect of imeglimin.

Therefore, metformin through modulation of adipocytokines and PPARα/AMPK signaling pathway could be effective in MS management.

## 6 Interaction of Metformin With FGF21 in MS

Of note, FGF21 is highly down‐regulated in MS (Su et al. 2006); therefore, restoring FGF21 levels by metformin administration could be a therapeutic strategy in the management of MS. Metformin has been shown to increase the expression of hepatocyte FGF21 via AMPK‐dependent pathway in rats (Nygaard et al. 2012). FGF21 mRNA and protein expression are reduced in both rat and human hepatocytes treated with metformin when incubated with Compound C (an AMPK inhibitor) (Nygaard et al. 2012). Therefore, metformin is a potent inducer of hepatic FGF21 expression and that the effect of metformin seems to be mediated through AMPK activation. In addition, activated FGF21 enhances the expression of AMPK, which regulates mitochondrial function (Md 2010). Consequently, both FGF21 and metformin accelerate the expression of AMPK, a critical factor in the remyelination process in MS (Largani et al. 2019). In addition, FGF21 promotes the expression of SIRTI and PGC‐1α signaling pathways (Md 2010), which are down‐regulated in MS (Li et al. 2020; Witte et al. 2013). This dual activation by metformin and FGF21 suggests a synergistic potential in modulating mitochondrial function and promoting remyelination, making it a promising approach in the treatment of MS.

### 6.1 SIRTI and PGC‐1α

SIRTI regulates various signal transductions to control gene expression involved in the regulation of energy metabolism, inflammation, and oxidative stress during neuronal injury (Li et al. 2020). SIRTI signaling is reduced in different neurological disorders, including MS (Li et al. 2020; Sharma et al. 2021). SIRTI dysregulation affects transcription factors, and other molecular alterations such as gene expression modification influence neuronal plasticity inhibit Th17 cells, and interleukin‐1β can aggravate brain diseases. However, up‐regulation of SIRT‐1 reduces autoimmunity, neurodegeneration, and neuroexcitation (Sharma et al. 2021). The expression of SIRT1 in MS brains and in peripheral blood mononuclear cells (PBMCs) obtained from patients with RRMS was significant decrease during relapses when compared with the levels in controls and stable MS patients (Tegla et al. 2014). It has been shown that genetic variations in *SIRT1* rs3818292, rs3758391, and rs7895833 are related with MS, with possible differences in gender and age, as well as lower serum SIRT1 levels (Kaikaryte et al. 2023). Thus, SIRT1 may represent a biomarker of relapses and understanding the SIRT‐1 signaling and identifying immune‐mediated neuron deterioration can detect major therapeutic interventions that could prevent neuro complications in MS. Similarly, PGC‐1α, a master regulator of mitochondrial function, is highly reduced in pyramidal neurons of the MS cortex (Witte et al. 2013). Reduction of PGC‐1α enhances the development of oxidative stress and mitochondrial dysfunction in the frontal cortex of MS patients (Witte et al. 2013). Recent proof proposes that ROS produced by inflammatory cells drive axonal degeneration in active MS lesions by inducing mitochondrial dysfunction. Mitochondria are endowed with a variety of antioxidant enzymes, including peroxiredoxin‐3, thioredoxin‐2 and PGC‐1α, which are involved in limiting ROS‐induced damage. Immunohistochemical analysis of a large cohort of MS patients discovered a prominent up‐regulation of PGC‐1α and downstream mitochondrial antioxidants in active demyelinating MS lesions. Enhanced expression was predominantly observed in reactive astrocytes as a compensatory mechanism (Nijland et al. 2014). Intriguingly, neuronal cells co‐cultured with PGC‐1α‐overexpressing astrocytes were protected against an exogenous oxidative attack compared to neuronal cells co‐cultured with control astrocytes. Enhanced astrocytic PGC‐1α levels markedly reduced the production and secretion of the pro‐inflammatory mediators IL‐6 and chemokine (Nijland et al. 2014). Thus, increased astrocytic PGC‐1α in active MS lesions might initially function as an endogenous protective mechanism to dampen oxidative damage and inflammation thereby reducing neurodegeneration. Activation of PGC‐1α therefore represents a promising therapeutic strategy to improve mitochondrial function and repress inflammation. In addition, up‐regulation of neuronal PGC‐1α protected neurons from apoptosis in EAE mouse model (Dang et al. 2019).

Notably, metformin promotes the expression of neuroprotective PGC‐1α and SIRT1 signaling (Aatsinki et al. 2014; Ren et al. 2020). Metformin promotes the expression of hepatic PGC‐1α in mice (Duan et al. 2019) and SIRTI signaling in diabetic rats (Ren et al. 2020). Findings from preclinical study illustrated that metformin protected dopaminergic neurons and improved dopamine‐sensitive motor performance in an MPTP‐induced PD animal model via ATF2/CREB‐PGC‐1α pathway (Kang et al. 2017). Likewise, metformin protects oligodendrocytes via activation of AMPK/PGC‐1α signaling pathway in mixed glial cultures (Paintlia et al. 2013). Furthermore, metformin improves mitophagy in neurodegenerative diseases by activating neuronal SIRT1 signaling (Chen et al. 2021). Besides, FGF21 increases the expression of neuronal PGC‐1α and SIRTI (Fang et al. 2025; Katsu‐Jiménez and Giménez‐Cassina 2019). Thus, metformin increases the expression of PGC‐1α and SIRTI signaling either directly or indirectly through activation of FGF21. This dual mechanism suggests that metformin could be beneficial in mitigating the neuropathology of MS by enhancing mitochondrial function and reducing oxidative stress and inflammation.

### 6.2 Glutamate‐Induced Excitotoxicity

Glutamate‐induced excitotoxicity and associated oxidative stress are linked with oligodendrocytes injury and neurodegeneration in MS (Xu et al. 2021; Gardón et al. 2022). It has been shown that the interaction between metformin and FGF21 is highly beneficial in mitigating neuronal injury induced by oxidative stress and glutamate‐induced excitotoxicity (Kar et al. 2022). Of note, FGF21 defends neurons from glutamate‐induced excitotoxicity by inhibiting oxidative and inflammatory reactions (Linares et al. 2020). FGF‐21 plays a crucial role in protecting mesenchymal stem cells from apoptosis induced by oxidative stress and inflammation (Linares et al. 2020). Furthermore, metformin attenuates LPS‐induced oxidative stress and inflammation by increasing the expression of FGF21 (Xu et al. 2021). Various studies highlighted the potential of metformin in amelioration oxidative stress and neuronal injury across neurological disorders (Wang et al. 2020; Hassan et al. 2020). Besides, metformin attenuates glutamate‐induced excitotoxicity in experimental stroke model (Zhou et al. 2016). Metformin promotes neuronal viability in neurodegenerative disorders by directly inhibiting glutamate‐induced excitotoxicity and neurotoxicity (Zhou et al. 2016). In this state, metformin through induction expression of FGF21 can potentiate its neuroprotective effect against oxidative stress and glutamate‐induced excitotoxicity in MS.

### 6.3 β‐Klotho

β‐Klotho, a transmembrane protein, functions as a co‐receptor for FGF21 (Yu 2015), contributing to its neuroprotective effects against MS (Kuroda et al. 2017). Mechanistically, leakage of circulating FGF21 which is predominantly expressed by the pancreas drives proliferation of oligodendrocyte precursor cells through interactions with β‐Klotho, an essential coreceptor of FGF21. Notably, human oligodendrocyte precursor cells expressed β‐Klotho and proliferated in response to FGF21 in vitro (Kuroda et al. 2017). It has been shown that elevating the expression of β‐Klotho could potentially enhance the neuroprotective effects of FGF21. An experimental study demonstrated that diabetic mice exhibited reduced FGF21 sensitivity due to elevated levels of circulating miR34a (Majeed et al. 2016). Up‐regulation of FGF‐21 and β‐Klotho in livers with a concomitant reduction in its downstream effectors, ERK and Sirt1 may lead to compromised FGF‐21 sensitivity (Majeed et al. 2016). Interestingly, RNA binding motif 3 (RBM3) is a powerful neuroprotectant that inhibits neurodegenerative cell death in vivo and is a promising therapeutic target in brain ischemia. RBM3 is increased by the FGF21 in rat cortical neurons. FGF21 receptor binding is controlled by the transmembrane protein β‐Klotho, which is mostly absent in the adult brain. However, RBM3/β‐Klotho is unexpectedly high in the human infant vs. adult brain (hippocampus/prefrontal cortex). RBM3/β‐Klotho is enriched in neurons in the developing brain (Jackson et al. 2019). Therefore, β‐Klotho has a neuroprotective effect against MS, and activating of this co‐receptor could be a therapeutic strategy in MS. It has been shown that metformin increase β‐Klotho and its downstream effectors, SIRT1 and ERK, thereby improving FGF21 sensitivity (Majeed et al. 2016). However, overexpression of FGF21 in T2DM can lead to FGF21 resistance (So and Leung 2016). In addition, IR and elevated levels of pro‐inflammatory cytokines such as TNF‐α suppress β‐Klotho expression, resulting in FGF21 resistance in adipocytes and exacerbating inflammatory disorders (Díaz‐Delfín et al. 2012). Moreover, recombinant human FGF21 (rhFGF21) activates PPARγ in TNF‐α‐induced HBMECs through formation of an FGF21/FGFR1/β‐Klotho complex which up‐regulated TJ and AJ proteins (Chen et al. 2018). Therefore, FGF21 by protecting of the BBB attenuates the progression of MS neuropathology. Thus, metformin alleviates IR by regulate β‐Klotho expression and counteract FGF21 resistance. These findings suggest that metformin may enhance FGF21's functional activity against the development and progression of MS by increasing β‐Klotho expression.

### 6.4 Immune Response

FGF21 inhibits the activity of T lymphocytes and the expression of pro‐inflammatory cytokines (Singhal et al. 2016), and blocks NF‐κB, a master regulator of B and T lymphocytes (Yu et al. 2016). In vitro, FGF21 reduced the expression of TNF‐α, IL‐1β, IL‐6, and IFN‐γ and increased the level of IL‐10 in a dose‐dependent manner in LPS‐stimulated RAW 264.7 macrophages. FGF21 also suppresses ROS production and oxidative stress by restoring the activities of antioxidant enzymes in LPS‐stimulated RAW 264.7 macrophages. Furthermore, FGF21 inhibits LPS‐induced NF‐κB activation, including degradation of I‐κB and nuclear translocation of p65. As well, FGF21 induced heme oxygenase‐1 (HO‐1) expression and increased the nuclear transcription factor‐E2‐related factor 2 (Nrf2) levels in a dose‐dependent manner in LPS‐stimulated RAW 264.7 macrophages (Yu et al. 2016). Thus, FGF21 exerts an anti‐inflammatory effect by enhancing Nrf2‐mediated antioxidant capacity and suppressing NF‐κB signaling pathway. Accordingly, FGF21 attenuates the immunoinflammatory response in MS neuropathology. The augmentation of FGF21 expression by metformin further inhibits autoreactive T cells in MS (Nath et al. 2009). Metformin also inhibits T cell‐mediated immune responses by inducing the expression of FGF21 mRNA in the brain of EAE model (Nath et al. 2009). Furthermore, metformin, through an AMPK‐dependent mechanism, enhances anti‐inflammatory regulatory T and inhibits the progression of the Th1 and Th17 immune response (Duan et al. 2019). Remarkably, the inhibition of glucose metabolism suppresses T helper lymphocytes involved in the production of autoantibodies. Therefore, the modulation of glucose metabolism by metformin can eliminate autoreactive T helper lymphocytes and prevent the development of systemic autoimmunity (Choi et al. 2018). In addition, metformin inhibits systemic autoimmunity in mice by suppressing the differentiation of B lymphocytes into plasma cells (Lee et al. 2017). Likewise, FGF21 can block the differentiation of B lymphocytes into plasma cells (Wan 2013), a hallmark in the pathogenesis of MS. Thus, metformin, through activation of FGF21, may inhibit early pathogenic mechanism involved in the pathogenesis of MS.

### 6.5 Homeostatic Pathway

Of not, disruption of the BBB integrity in MS facilitates the entry of clotting factors, such as fibrinogen, into the CNS with subsequent induction of neuroinflammation (Yates et al. 2017). A cohort study, involved 47 postmortem brains of MS patients and 10 healthy controls, revealed significantly a higher fibrinogen deposition in the motor cortex of MS patients compared with controls (Yates et al. 2017). This pathological feature arises from deregulation of fibrinolysis and increased fibrinogen‐induced neurotoxicity in MS (Yates et al. 2017). In addition, fibrinogen is implicated in MS neuropathology through the activation of microglia (Adams et al. 2007). Perivascular microglia activation is a hallmark of inflammatory demyelination in MS, but the mechanisms underlying microglia activation and specific strategies to attenuate their activation remain elusive. It has been reported that fibrinogen as a novel regulator of microglia activation and targeting of the interaction of fibrinogen with the microglia integrin receptor Mac‐1 is adequate to suppress EAE in mice. Fibrinogen, which is deposited in MS plaques, signals through Mac‐1 can induces the differentiation of microglia to phagocytes via activation of Akt and Rho. Genetic disruption of fibrinogen‐Mac‐1 interaction in fibrinogen‐gamma (390‐396A) knock‐in mice or pharmacologically impeding fibrinogen‐Mac‐1 interaction through intranasal delivery of a fibrinogen‐derived inhibitory peptide attenuates microglia activation and suppresses relapsing paralysis (Adams et al. 2007). Therefore, targeting fibrinogen or the fibrinolysis pathway could reduce MS neuropathology. Interestingly, FGF21 has been reported to inhibit thrombus formation without increasing the risk of bleeding (Li et al. 2022). This finding indicates that FGF21 has antithrombotic effects by inhibiting the expression of fibrinogen and platelet aggregation while it enhancing fibrinolysis by activating the expression of tissue plasminogen activator (tPA) and inhibition of plasminogen activator inhibitor 1(tPA‐1) (Li et al. 2022). As well, FGF21 inhibits thrombosis‐induced inflammation by blocking the NF‐κB signaling pathway (Li et al. 2022). Recently, tPA and tPA‐1 are dysregulated in MS patients (Abbadessa et al. 2022). Therefore, activation of FGF21 signaling may mitigate MS neuropathology by regulating the homeostatic pathway. Besides, metformin, regarded as a potent activator of FGF21, plays a critical role in the regulation of blood homeostasis. Several studies have reported the fibrinolytic effects of metformin (Krysiak and Okopien 2012; Serdyńska‐Szuster et al. 2011). In vivo and ex vivo studies illustrated that metformin reduces circulating fibrinogen and PAI‐1, interferes with fibrin polymerization, and promotes clot‐lysis effect (Standeven et al. 2002; Grant 2003).

Moreover, endothelial dysfunction and platelet dysfunction contribute to the progression and development of neurodegenerative diseases, including MS (Ahmad et al. 2020). The interaction between platelets and immune cells triggers neurovascular inflammation (Ahmad et al. 2020). Notably, platelet granules and mediators such as β thromboglobulin (βTG) and platelet factor 4 (PF‐4) are activated in MS (Dziedzic and Bijak 2019; Wachowicz et al. 2016). The interaction between platelets and leukocytes promotes neuroinflammation in MS (Dziedzic and Bijak 2019). Similarly, the interaction between platelets and immune cells, along with the release of platelet microparticles, is involved in the development of autoimmune encephalomyelitis (Wachowicz et al. 2016). PF‐4 enhances the differentiation of monocytes into macrophages, causing inflammation and oxidative stress (Y. Zhang et al. 2013). FGF21 has been shown to effectively reduce thromboembolic disorders by inhibiting platelet activation (Takeda et al. 2015). The increase of FGF21 in ischemic stroke could be a compensatory mechanism to inhibit platelet aggregation (Maglinger et al. 2021). Besides, metformin and its derivatives have a potential antiplatelet effect and help maintain platelet homeostasis (Wijnen et al. 2014; Markowicz‐Piasecka et al. 2019). Moreover, metformin reduces the expression of platelet receptors and activators like αIIbβ3 and P‐selectin (Xin et al. 2016). Likewise, metformin decreases the risk of thrombosis by inhibiting oxidative stress and release of extracellular mitochondrial DNA (Nijland et al. 2014). Metformin prevents the development of platelet mitochondrial dysfunction by regulating Ca^2+^ homeostasis (Markowicz‐Piasecka et al. 2019; Xin et al. 2016). Therefore, modulation of platelet function and coagulation cascades by FGF21 and its activators like metformin may mitigate thrombotic‐induced MS neuropathology. Taken together, metformin, through activation of FGF21, and related signaling pathways can mitigate different neuropathological processes in MS (Table 1).

**TABLE 1:** Roles of metformin and FGF21in MS.

Study type	Findings	Ref.
Preclinical	FGF21 mRNA and protein expression are reduced in both rat and human hepatocytes treated with AMPK inhibitor.	(Nygaard et al. 2012).
Preclinical	FGF21 and metformin accelerate the expression of AMPK, a critical factor in the remyelination process in MS.	(Largani et al. 2019).
Preclinical	Metformin promotes the expression of neuroprotective PGC‐1α and SIRT1 signaling.	(Aatsinki et al. 2014; Ren et al. 2020).
Preclinical	Metformin protects dopaminergic neurons and improved dopamine‐sensitive motor performance in an MPTP‐induced PD animal model via ATF2/CREB‐PGC‐1α pathway.	(Kang et al. 2017).
Preclinical	Metformin protects oligodendrocytes via activation of AMPK/PGC‐1α signaling pathway in mixed glial cultures.	(Paintlia et al. 2013).
Preclinical	Metformin improves mitophagy in neurodegenerative diseases by activating neuronal SIRT1 signaling.	(Chen et al. 2021).
Preclinical	FGF‐21 protects mesenchymal stem cells from apoptosis induced by oxidative stress and inflammation.	(Linares et al. 2020).
Preclinical	Metformin attenuates LPS‐induced oxidative stress and inflammation by increasing the expression of FGF21.	(Xu et al. 2021).
Preclinical	Metformin attenuates glutamate‐induced excitotoxicity in experimental stroke model by inhibiting glutamate‐induced excitotoxicity and neurotoxicity.	(Zhou et al. 2016).
Preclinical	Metformin increases β‐Klotho and its downstream effectors, SIRT1 and ERK, thereby improving FGF21 sensitivity.	(Majeed et al. 2016).
Preclinical	FGF21 by protecting of the BBB attenuates the progression of MS neuropathology.	(Chen et al. 2018).
Preclinical	Metformin also inhibits T cell‐mediated immune responses by inducing the expression of FGF21 mRNA in the brain of EAE model.	(Nath et al. 2009).

The present review has several limitations, including that most of findings were from preclinical studies that does not specifically addressing the role of FGF21 in MS patients. In addition, biomarkers of metformin and FGF21 effects in MS have not been assessed in clinical studies. A key strength of this review is the suggestion of metformin's potential impact on MS neuropathology through the augmentation of the neuroprotective FGF21. Therefore, this review highlights the need for future clinical trials and pilot studies to confirm the potential role of metformin in the development and progression of MS through FGF21 signaling.

## 7 Conclusions

MS is a progressive demyelinating disease of the CNS characterized by immune‐mediated injury of the myelin sheath. The MS neuropathology involves the formation of CNS plaques, inflammation, and damage to the neuronal myelin sheath that induced by both genetic and environmental factors. MS is classified as an immune‐mediated disease triggered by the hyperactivation of peripheral autoreactive T lymphocytes, which induce inflammatory changes within the CNS. FGF21, a growth factor involved in different metabolic disorders and neurodegenerative disorders, exhibits neuroprotective effects against neurodegenerative diseases through inhibition of mitochondrial dysfunction, cerebrovascular aging, and associated inflammatory and oxidative stress disorders. FGF21 promotes the differentiation and proliferation of oligodendrocyte precursor cells, which are essential for the remyelination process in MS. FGF21 and its co‐receptor β‐Kloth play crucial roles in the remyelination of MS.

Activation of FGF21 by metformin can mitigates the pathogenesis of MS. Metformin in virtue of its anti‐inflammatory could be effective against MS neuropathology. Metformin also influences the expression of FGF21 and attenuates the inflammatory reactions in MS. It increases the expression of PGC‐1α and SIRTI signaling, either directly or indirectly, through the activation of FGF21. Metformin enhances β‐Klotho expression, modulates oxidative stress, reduces glutamate‐induced excitotoxicity, and regulates platelet function and coagulation cascades.

These observations suggest that metformin can improve the functional activity of FGF21 in counteracting the development and progression of MS. Preclinical and clinical studies are warranted to investigate these effects further and confirm the potential therapeutic role of metformin in MS through FGF21 signaling.

## Author Contributions

**Ahmad A. Abulaban:** conceptualization, resources, writing – original draft, writing – review and editing. **Hayder M. Al‐kuraishy:** conceptualization, writing – original draft, writing – review and editing. **Ali I. Al‐Gareeb:** conceptualization, writing – original draft, writing – review and editing. **Eman abdelnaby Ahmed:** conceptualization, visualization, writing – original draft, writing – review and editing. **Mubarak Alruwaili:** conceptualization, resources, writing – original draft, writing – review and editing. **Athanasios Alexiou:** conceptualization, data curation, writing – original draft. **Marios Papadakis:** supervision, writing – original draft, writing – review and editing. **Gaber El‐Saber Batiha:** supervision, writing – original draft, writing – review and editing.

## Ethics Statement

The authors have nothing to report.

## Consent

The authors have nothing to report.

## Conflicts of Interest

The authors declare no conflicts of interest.

### Peer Review

The peer review history for this article is available at https://www.webofscience.com/api/gateway/wos/peer‐review/10.1111/ejn.70067.

## Data Availability

The authors have nothing to report.

## References

[ejn70067-bib-0001] Aatsinki, S. M. , M. Buler , H. Salomäki , M. Koulu , P. Pavek , and J. Hakkola . 2014. “Metformin Induces PGC‐1α Expression and Selectively Affects Hepatic PGC‐1α Functions.” British Journal of Pharmacology 171, no. 9: 2351–2363. 24428821 10.1111/bph.12585 PMC3997275

[ejn70067-bib-0002] Abbadessa, G. , L. Lavorgna , C. A. Treaba , S. Bonavita , and C. Mainero . 2022. “Hemostatic Factors in the Pathogenesis of Neuroinflammation in Multiple Sclerosis.” Multiple Sclerosis Journal 28, no. 12: 1834–1842. 34410198 10.1177/13524585211039111

[ejn70067-bib-0003] Abdul‐Hadi, M. H. , M. T. Naji , H. A. Shams , O. M. Sami , H. M. Al‐Kuraishy , and A. I. Al‐Gareeb . 2020. “Erectile Dysfunction and Type 2 Diabetes Mellitus: A New Twist.” International Journal of Nutrition, Pharmacology, Neurological Diseases 10, no. 2: 43–49.

[ejn70067-bib-0004] Abdul‐Hadi, M. H. , M. T. Naji , H. A. Shams , et al. 2020. “Oxidative Stress Injury and Glucolipotoxicity in Type 2 Diabetes Mellitus: The Potential Role of Metformin and Sitagliptin.” Biomedical and Biotechnology Research Journal (BBRJ) 4, no. 2: 166–172.

[ejn70067-bib-0005] Adams, A. C. , C. C. Cheng , T. Coskun , and A. Kharitonenkov . 2012. “FGF21 Requires βklotho to Act In Vivo.” PLoS ONE 7, no. 11: e49977. 23209629 10.1371/journal.pone.0049977 PMC3507945

[ejn70067-bib-0006] Adams, R. A. , J. Bauer , M. J. Flick , et al. 2007. “The Fibrin‐Derived γ377‐395 Peptide Inhibits Microglia Activation and Suppresses Relapsing Paralysis in Central Nervous System Autoimmune Disease.” Journal of Experimental Medicine 204, no. 3: 571–582. 17339406 10.1084/jem.20061931 PMC2137908

[ejn70067-bib-0007] Ahmad, A. , V. Patel , J. Xiao , and M. M. Khan . 2020. “The Role of Neurovascular System in Neurodegenerative Diseases.” Molecular Neurobiology 57: 4373–4393. 32725516 10.1007/s12035-020-02023-z

[ejn70067-bib-0008] Aleagha, M. S. E. , B. Siroos , M. Ahmadi , et al. 2015. “Decreased Concentration of Klotho in the Cerebrospinal Fluid of Patients With Relapsing–Remitting Multiple Sclerosis.” Journal of Neuroimmunology 281: 5–8. 25867461 10.1016/j.jneuroim.2015.02.004

[ejn70067-bib-0009] Alhossan, A. , N. F. Alaifan , B. T. Althwaini , and A. Ahmad . 2022. “Evaluation of Antipyretics Use and Heat Sensitivity in Patients With Multiple Sclerosis and Its Impact On Qol.” Farmácia 70, no. 4: 704–711.

[ejn70067-bib-0010] Al‐Kuraishy, H. M. , and A. I. Al‐Gareeb . 2016. “Erectile Dysfunction and Low Sex Drive in Men With Type 2 DM: The Potential Role of Diabetic Pharmacotherapy.” Journal of Clinical and Diagnostic Research: JCDR 10, no. 12: FC21. 28208875 10.7860/JCDR/2016/19971.8996 PMC5296448

[ejn70067-bib-0011] Al‐Kuraishy, H. M. , and A. I. Al‐Gareeb . 2016. “Effect of Orlistat Alone or in Combination With Garcinia Cambogia on Visceral Adiposity Index in Obese Patients.” Journal of Intercultural Ethnopharmacology 5, no. 4: 408. 27757272 10.5455/jice.20160815080732 PMC5061485

[ejn70067-bib-0012] Al‐Kuraishy, H. M. , A. I. Al‐Gareeb , S. M. Abdullah , N. Cruz‐Martins , and G. E. Batiha . 2021. “Case Report: Hyperbilirubinemia in Gilbert Syndrome Attenuates Covid‐19‐Induced Metabolic Disturbances.” Frontiers in Cardiovascular Medicine 8: 642181. 33681310 10.3389/fcvm.2021.642181 PMC7925614

[ejn70067-bib-0013] Al‐Kuraishy, H. M. , A. I. Al‐Gareeb , M. Alblihed , N. Cruz‐Martins , and G. E.‐S. Batiha . 2021. “COVID‐19 and Risk of Acute Ischemic Stroke and Acute Lung Injury in Patients With Type ii Diabetes Mellitus: The Anti‐Inflammatory Role of Metformin.” Frontiers in Medicine 8: 644295. 33718411 10.3389/fmed.2021.644295 PMC7944640

[ejn70067-bib-0014] Al‐Kuraishy, H. M. , A. I. Al‐Gareeb , M. Alblihed , S. G. Guerreiro , N. Cruz‐Martins , and G. E.‐S. Batiha . 2021. “COVID‐19 in Relation to Hyperglycemia and Diabetes Mellitus.” Frontiers in Cardiovascular Medicine 8: 644095. 34124187 10.3389/fcvm.2021.644095 PMC8189260

[ejn70067-bib-0015] Al‐Kuraishy, H. M. , A. I. Al‐Gareeb , S. M. Albogami , et al. 2022. “Potential Therapeutic Benefits of Metformin Alone and in Combination With Sitagliptin in the Management of Type 2 Diabetes Patients With COVID‐19.” Pharmaceuticals 15, no. 11: 1361. 36355535 10.3390/ph15111361 PMC9699540

[ejn70067-bib-0016] Al‐Kuraishy, H. M. , A. I. Al‐Gareeb , A. Alexiou , et al. 2022. “Metformin and Growth Differentiation Factor 15 (GDF15) in Type 2 Diabetes Mellitus: A Hidden Treasure.” Journal of Diabetes 14, no. 12: 806–814. 10.1111/1753-0407.13334. 36444166 PMC9789395

[ejn70067-bib-0017] Al‐Kuraishy, H. M. , A. I. Al‐Gareeb , M. S. Al‐Niemi , A. K. Al‐Buhadily , N. A. Al‐Harchan , and C. Lugnier . 2020. “COVID‐19 and Phosphodiesterase Enzyme Type 5 Inhibitors.” Journal of Microscopy and Ultrastructure 8, no. 4: 141–145. 33623736 10.4103/JMAU.JMAU_63_20 PMC7883493

[ejn70067-bib-0018] Al‐Kuraishy, H. M. , A. I. Al‐Gareeb , M. S. Al‐Niemi , et al. 2022. “The Prospective Effect of Allopurinol on the Oxidative Stress Index and Endothelial Dysfunction in Covid‐19.” Inflammation 45, no. 4: 1651–1667. 35199285 10.1007/s10753-022-01648-7 PMC8865950

[ejn70067-bib-0019] Al‐Kuraishy, H. M. , A. I. Al‐Gareeb , M. Alqarni , N. Cruz‐Martins , and G. El‐Saber Batiha . 2021. “Pleiotropic Effects of Tetracyclines in the Management of COVID‐19: Emerging Perspectives.” Frontiers in Pharmacology 12: 642822. 33967777 10.3389/fphar.2021.642822 PMC8103613

[ejn70067-bib-0020] Al‐Kuraishy, H. M. , A. I. Al‐Gareeb , A. A. Alsayegh , et al. 2023. “A Potential Link Between Visceral Obesity and Risk of Alzheimer's Disease.” Neurochemical Research 48, no. 3: 745–766. 36409447 10.1007/s11064-022-03817-4

[ejn70067-bib-0022] Al‐kuraishy, H. M. , A. I. Al‐Gareeb , and S. A. Al‐Windy . 2016. “Evaluation the Effect of Glyburide and/or Metformin on Testosterone Levels in Men Patients With Type 2 Diabetes Mellitus.” Age (Years) 40: 60.

[ejn70067-bib-0023] Al‐Kuraishy, H. M. , A. I. Al‐Gareeb , and G. E. Batiha . 2022. “The Possible Role of Ursolic Acid in Covid‐19: A Real Game Changer.” Clinical Nutrition ESPEN 47: 414–417. 35063236 10.1016/j.clnesp.2021.12.030 PMC8724013

[ejn70067-bib-0024] Al‐Kuraishy, H. M. , A. I. Al‐Gareeb , E. Elekhnawy , A. Alexiou , and G. E. Batiha . 2024. “The Potential Effect of Dapsone on the Inflammatory Reactions in COVID‐19: Staggering View.” Combinatorial Chemistry & High Throughput Screening 27, no. 5: 674–678. 36999691 10.2174/1386207326666230331121735

[ejn70067-bib-0025] Al‐Kuraishy, H. M. , A. I. Al‐Gareeb , E. Elekhnawy , and G. E. Batiha . 2022. “Nitazoxanide and COVID‐19: A Review.” Molecular Biology Reports 49, no. 11: 11169–11176. 36094778 10.1007/s11033-022-07822-2 PMC9465141

[ejn70067-bib-0026] Al‐kuraishy, H. M. , A. I. Al‐Gareeb , M. S. Jabir , and S. Albukhaty . 2023. “Effects of Metformin on Fibroblast Growth Factor 21 in Patients With Type 2 Diabetes Mellitus: Faraway but so Close.” Egyptian Journal of Internal Medicine 35, no. 1: 65.

[ejn70067-bib-0027] Al‐Kuraishy, H. M. , A. I. Al‐Gareeb , S. Qusti , E. M. Alshammari , F. O. Atanu , and G. E. Batiha . 2021. “Arginine Vasopressin and Pathophysiology of COVID‐19: An Innovative Perspective.” Biomedicine & Pharmacotherapy 143: 112193. 34543987 10.1016/j.biopha.2021.112193 PMC8440235

[ejn70067-bib-0028] Al‐Kuraishy, H. M. , A. I. Al‐Gareeb , N. Qusty , A. Alexiou , and G. E. Batiha . 2022. “Impact of Sitagliptin on Non‐Diabetic Covid‐19 Patients.” Current Molecular Pharmacology 15, no. 4: 683–692. 34477540 10.2174/1874467214666210902115650

[ejn70067-bib-0029] Al‐Kuraishy, H. M. , A. I. Al‐Gareeb , H. M. Saad , and G. E. Batiha . 2023a. “The Potential Therapeutic Effect of Statins in Multiple Sclerosis: Beneficial or Detrimental Effects.” Inflammopharmacology 31, no. 4: 1671–1682. 37160526 10.1007/s10787-023-01240-x

[ejn70067-bib-0030] Al‐Kuraishy, H. M. , A. I. Al‐Gareeb , H. M. Saad , and G. E. Batiha . 2023b. “The Potential Effect of Metformin on Fibroblast Growth Factor 21 in Type 2 Diabetes Mellitus (T2DM).” Inflammopharmacology 31, no. 4: 1751–1760. 37337094 10.1007/s10787-023-01255-4

[ejn70067-bib-0031] Al‐Kuraishy, H. M. , A. I. Al‐Gareeb , H. M. Saad , and G. E. Batiha . 2023c. “Long‐Term Use of Metformin and Alzheimer's Disease: Beneficial or Detrimental Effects.” Inflammopharmacology 31, no. 3: 1107–1115. 36849855 10.1007/s10787-023-01163-7

[ejn70067-bib-0032] Al‐Kuraishy, H. M. , A. I. Al‐Gareeb , H. A. Shams , and F. Al‐Mamorri . 2019. “Endothelial Dysfunction and Inflammatory Biomarkers as a Response Factor of Concurrent Coenzyme Q10 Add‐On Metformin in Patients With Type 2 Diabetes Mellitus.” Journal of Laboratory Physicians 11, no. 04: 317–322. 31929697 10.4103/JLP.JLP_123_19 PMC6943859

[ejn70067-bib-0033] Alkuraishy, H. M. , A. I. Al‐Gareeb , and H. J. Waheed . 2018 Oct. “Lipoprotein‐Associated Phospholipase A2 Is Linked With Poor Cardio‐Metabolic Profile in Patients With Ischemic Stroke: A Study of Effects of Statins.” Journal of Neurosciences in Rural Practice 9, no. 4: 496–503. 30271040 10.4103/jnrp.jnrp_97_18 PMC6126307

[ejn70067-bib-0034] Al‐Kuraishy, H. M. , A. I. Al‐Gareeb , H. J. Waheed , and T. J. Al‐Maiahy . 2018. “Differential Effect of Metformin and/or Glyburide on Apelin Serum Levels in Patients With Type 2 Diabetes Mellitus: Concepts and Clinical Practice.” Journal of Advanced Pharmaceutical Technology & Research 9, no. 3: 80–86. 30338233 10.4103/japtr.JAPTR_273_18 PMC6174705

[ejn70067-bib-0035] Al‐Kuraishy, H. M. , T. J. Al‐Maiahy , A. I. Al‐Gareeb , R. A. Musa , and Z. H. Ali . 2020. “COVID‐19 Pneumonia in an Iraqi Pregnant Woman With Preterm Delivery.” Asian Pacific Journal of Reproduction 9, no. 3: 156–158.

[ejn70067-bib-0036] Al‐Kuraishy, H. M. , M. S. Al‐Naimi , C. M. Lungnier , and A. I. Al‐Gareeb . 2020. “Macrolides and COVID‐19: An Optimum Premise.” Biomedical and Biotechnology Research Journal (BBRJ) 4, no. 3: 189–192.

[ejn70067-bib-0037] Al‐Kuraishy, H. M. , M. T. Hamada , and A. Y. Al‐Samerraie . 2016. “Effects of Metformin on Omentin‐1 Serum Levels in a Newly Diagnosed Type 2 Diabetes Mellitus: Randomized, Placebo Controlled Study.” Mustansiriya Medical Journal 15, no. 1: 49–56.

[ejn70067-bib-0038] Al‐Kuraishy, H. M. , N. R. Hussien , M. S. Al‐Naimi , A. K. Al‐Buhadily , A. I. Al‐Gareeb , and C. Lungnier . 2020. “Is Ivermectin–Azithromycin Combination the Next Step for COVID‐19?” Biomedical and Biotechnology Research Journal (BBRJ) 4, no. Suppl 1: S101–S103.

[ejn70067-bib-0039] Al‐Kuraishy, H. M. , M. S. Jabir , A. I. Al‐Gareeb , H. M. Saad , G. E. Batiha , and D. J. Klionsky . 2024. “The Beneficial Role of Autophagy in Multiple Sclerosis: Yes or No?” Autophagy 20, no. 2: 259–274. 37712858 10.1080/15548627.2023.2259281 PMC10813579

[ejn70067-bib-0040] Al‐Kuraishy, H. M. , O. M. Sami , N. R. Hussain , and A. I. Al‐Gareeb . 2020. “Metformin and/or Vildagliptin Mitigate Type II Diabetes Mellitus Induced‐Oxidative Stress: The Intriguing Effect.” Journal of Advanced Pharmaceutical Technology & Research 11, no. 3: 142–147. 33102198 10.4103/japtr.JAPTR_18_20 PMC7574736

[ejn70067-bib-0041] Al‐Kuraishy, H. M. , G. M. Sulaiman , H. A. Mohammed , et al. 2024. “The Compelling Role of Brain‐Derived Neurotrophic Factor Signaling in Multiple Sclerosis: Role of BDNF Activators.” CNS Neuroscience & Therapeutics 30, no. 12: e70167. 39654365 10.1111/cns.70167 PMC11628746

[ejn70067-bib-0042] Alnaaim, S. A. , H. M. Al‐kuraishy , A. I. Al‐Gareeb , et al. 2023. “New Insights on the Potential Anti‐Epileptic Effect of Metformin: Mechanistic Pathway.” Journal of Cellular and Molecular Medicine 27, no. 24: 3953–3965. 37737447 10.1111/jcmm.17965 PMC10747420

[ejn70067-bib-0043] Al‐Nami, M. S. , H. M. Al‐Kuraishy , and A. I. Al‐Gareeb . 2020. “Impact of Thioctic Acid on Glycemic Indices and Associated Inflammatory‐Induced Endothelial Dysfunction in Patients With Type 2 Diabetes Mellitus: A Case Control Study.” International Journal of Critical Illness and Injury Science 10, no. Suppl 1: 21–27. 33376686 10.4103/IJCIIS.IJCIIS_62_19 PMC7759067

[ejn70067-bib-0044] Aloisi, F. , and A. H. Cross . 2022. “MINI‐Review of Epstein‐Barr Virus Involvement in Multiple Sclerosis Etiology and Pathogenesis.” Journal of Neuroimmunology 371: 577935. 35931008 10.1016/j.jneuroim.2022.577935

[ejn70067-bib-0045] Alorabi, M. , S. Cavalu , H. M. Al‐Kuraishy , et al. 2022. “Pentoxifylline and Berberine Mitigate Diclofenac‐Induced Acute Nephrotoxicity in Male Rats via Modulation of Inflammation and Oxidative Stress.” Biomedicine & Pharmacotherapy 152: 113225. 35671584 10.1016/j.biopha.2022.113225

[ejn70067-bib-0046] Alrouji, M. , H. M. Al‐Kuraishy , A. I. Al‐Gareeb , et al. 2024. “Metformin Role in Parkinson's Disease: A Double‐Sword Effect.” Molecular and Cellular Biochemistry 479, no. 4: 975–991. 37266747 10.1007/s11010-023-04771-7

[ejn70067-bib-0047] Alruwaili, M. , H. M. Al‐Kuraishy , A. Alexiou , et al. 2023. “Pathogenic Role of Fibrinogen in the Neuropathology of Multiple Sclerosis: A Tale of Sorrows and Fears.” Neurochemical Research 48, no. 11: 3255–3269. 37442896 10.1007/s11064-023-03981-1 PMC10514123

[ejn70067-bib-0048] Alsubaie, N. , H. M. Al‐Kuraishy , A. I. Al‐Gareeb , et al. 2022. “Statins Use in Alzheimer Disease: Bane or Boon From Frantic Search and Narrative Review.” Brain Sciences 12, no. 10: 1290. 36291224 10.3390/brainsci12101290 PMC9599431

[ejn70067-bib-0049] Al‐Thomali, A. W. , H. M. Al‐Kuraishy , A. I. Al‐Gareeb , et al. 2022. “Role of Neuropilin 1 in COVID‐19 Patients With Acute Ischemic Stroke.” Biomedicine 10, no. 8: 2032. 10.3390/biomedicines10082032 PMC9405641 36009579

[ejn70067-bib-0050] Amiri, M. , N. Braidy , and M. Aminzadeh . 2018. “Protective Effects of Fibroblast Growth Factor 21 Against Amyloid‐Beta1–42‐Induced Toxicity in SH‐SY5Y Cells.” Neurotoxicity Research 34, no. 3: 574–583. 29869772 10.1007/s12640-018-9914-2

[ejn70067-bib-0051] Autonomic, A. J. M. 2003. “Nervous System Function in Multiple Sclerosis.” Journal of the Neurological Sciences 215: 79–85. 14568133 10.1016/s0022-510x(03)00205-3

[ejn70067-bib-0052] Babalghith, A. O. , H. M. Al‐Kuraishy , A. I. Al‐Gareeb , et al. 2022. “The Potential Role of Growth Differentiation Factor 15 in COVID‐19: A Corollary Subjective Effect or Not?” Diagnostics 12, no. 9: 2051. 36140453 10.3390/diagnostics12092051 PMC9497461

[ejn70067-bib-0053] Balasa, R. , L. Barcutean , A. Balasa , A. Motataianu , C. Roman‐Filip , and D. Manu . 2020. “The Action of TH17 Cells on Blood Brain Barrier in Multiple Sclerosis and Experimental Autoimmune Encephalomyelitis.” Human Immunology 81, no. 5: 237–243. 32122685 10.1016/j.humimm.2020.02.009

[ejn70067-bib-0054] Balkuv, E. , A. O. Varoglu , N. Isik , et al. 2016. “The Effects of Thrombomodulin and Activated Protein C on the Pathogenesis of Multiple Sclerosis.” Multiple Sclerosis and Related Disorders 8: 131–135. 10.1016/j.msard.2016.05.017. 27456888

[ejn70067-bib-0055] Batiha, G. E. , A. Gari , N. Elshony , et al. 2021. “Hypertension and Its Management in COVID‐19 Patients: The Assorted View.” International Journal of Cardiology. Cardiovascular Risk and Prevention 11: 200121. 34806090 10.1016/j.ijcrp.2021.200121 PMC8590508

[ejn70067-bib-0056] Batiha, G. E. , L. Wasef , J. O. Teibo , et al. 2023. “Commiphora Myrrh: A Phytochemical and Pharmacological Update.” Naunyn‐Schmiedeberg's Archives of Pharmacology 396, no. 3: 405–420. 36399185 10.1007/s00210-022-02325-0 PMC9672555

[ejn70067-bib-0057] BonDurant, L. D. , and M. J. Potthoff . 2018. “Fibroblast Growth Factor 21: A Versatile Regulator of Metabolic Homeostasis.” Annual Review of Nutrition 38, no. 1: 173–196. 10.1146/annurev-nutr-071816-064800 PMC6964258 29727594

[ejn70067-bib-0058] Buscarinu, M. C. , A. Fornasiero , S. Romano , et al. 2019. “The Contribution of gut Barrier Changes to Multiple Sclerosis Pathophysiology.” Frontiers in Immunology 10: 1916. 31555257 10.3389/fimmu.2019.01916 PMC6724505

[ejn70067-bib-0059] Chen, A. , C. K. Kristiansen , Y. Hong , et al. 2021. “Nicotinamide Riboside and Metformin Ameliorate Mitophagy Defect in Induced Pluripotent Stem Cell‐Derived Astrocytes With POLG Mutations.” Frontiers in Cell and Development Biology 9: 737304. 10.3389/fcell.2021.737304 PMC8497894 34631714

[ejn70067-bib-0060] Chen, F. , R. R. Dong , K. L. Zhong , et al. 2016. “Antidiabetic Drugs Restore Abnormal Transport of Amyloid‐β Across the Blood–Brain Barrier and Memory Impairment in db/db Mice.” Neuropharmacology 101: 123–136. 26211973 10.1016/j.neuropharm.2015.07.023

[ejn70067-bib-0061] Chen, J. , J. Hu , H. Liu , et al. 2018. “FGF21 Protects the Blood–Brain Barrier by Upregulating PPARγ via FGFR1/β‐Klotho After Traumatic Brain Injury.” Journal of Neurotrauma 35, no. 17: 2091–2103. 29648978 10.1089/neu.2017.5271

[ejn70067-bib-0063] Chen, S. , S.‐T. Chen , Y. Sun , et al. 2019. “Fibroblast Growth Factor 21 Ameliorates Neurodegeneration in Rat and Cellular Models of Alzheimer's Disease.” Redox Biology 22: 101133. 30785085 10.1016/j.redox.2019.101133 PMC6383137

[ejn70067-bib-0064] Choi, S.‐C. , A. A. Titov , G. Abboud , et al. 2018. “Inhibition of Glucose Metabolism Selectively Targets Autoreactive Follicular Helper T Cells.” Nature Communications 9, no. 1: 4369. 10.1038/s41467-018-06686-0. PMC6197193 30348969

[ejn70067-bib-0065] Coles, A. 2008. “Alastair Compston, Alasdair Coles.” Lancet 372: 1502–1517. 18970977 10.1016/S0140-6736(08)61620-7

[ejn70067-bib-0066] Cuevas‐Ramos, D. , P. Almeda‐Valdes , F. J. Gómez‐Pérez , et al. 2010. “Daily Physical Activity, Fasting Glucose, Uric Acid, and Body Mass Index Are Independent Factors Associated With Serum Fibroblast Growth Factor 21 Levels.” European Journal of Endocrinology 163, no. 3: 469–477. 20566587 10.1530/EJE-10-0454

[ejn70067-bib-0067] Dang, C. , B. Han , Q. Li , R. Han , and J. Hao . 2019. “Up‐Regulation of PGC‐1α in Neurons Protects Against Experimental Autoimmune Encephalomyelitis.” FASEB Journal 33, no. 12: 14811–14824. 31718280 10.1096/fj.201901149RR

[ejn70067-bib-0068] Derada Troletti, C. , R. D. Fontijn , E. Gowing , et al. 2019. “Inflammation‐Induced Endothelial to Mesenchymal Transition Promotes Brain Endothelial Cell Dysfunction and Occurs During Multiple Sclerosis Pathophysiology.” Cell Death & Disease 10, no. 2: 45. 30718504 10.1038/s41419-018-1294-2 PMC6361981

[ejn70067-bib-0069] Dërmaku‐Sopjania, M. , F. Kurtib , N. T. Xuanc , and M. Sopjanib . 2021. “Klotho‐Dependent Role of 1, 25 (OH).” Neuro‐Signals 29: 14–23. 33784444 10.33594/000000352

[ejn70067-bib-0070] Díaz‐Delfín, J. , E. Hondares , R. Iglesias , M. Giralt , C. Caelles , and F. Villarroya . 2012. “TNF‐α Represses β‐Klotho Expression and Impairs FGF21 Action in Adipose Cells: Involvement of JNK1 in the FGF21 Pathway.” Endocrinology 153, no. 9: 4238–4245. 22778214 10.1210/en.2012-1193

[ejn70067-bib-0071] Dludla, P. V. , B. B. Nkambule , S. E. Mazibuko‐Mbeje , et al. 2021. “Adipokines as a Therapeutic Target by Metformin to Improve Metabolic Function: A Systematic Review of Randomized Controlled Trials.” Pharmacological Research 163: 105219. 33017649 10.1016/j.phrs.2020.105219

[ejn70067-bib-0072] Dobson, R. , and G. Giovannoni . 2019. “Multiple Sclerosis–A Review.” European Journal of Neurology 26, no. 1: 27–40. 30300457 10.1111/ene.13819

[ejn70067-bib-0073] Douris, N. , D. M. Stevanovic , F. M. Fisher , et al. 2015. “Central Fibroblast Growth Factor 21 Browns White Fat via Sympathetic Action in Male Mice.” Endocrinology 156, no. 7: 2470–2481. 10.1210/en.2014-2001. 25924103 PMC4475718

[ejn70067-bib-0074] Duan, W. , Y. Ding , X. Yu , et al. 2019. “Metformin Mitigates Autoimmune Insulitis by Inhibiting Th1 and Th17 Responses While Promoting Treg Production.” American Journal of Translational Research 11, no. 4: 2393. 31105845 PMC6511786

[ejn70067-bib-0075] Dutta, R. , and B. D. Trapp . 2014. “Relapsing and Progressive Forms of Multiple Sclerosis: Insights From Pathology.” Current Opinion in Neurology 27, no. 3: 271–278. 24722325 10.1097/WCO.0000000000000094 PMC4132635

[ejn70067-bib-0076] Dyment, D. A. , G. C. Ebers , and A. D. Sadovnick . 2004. “Genetics of Multiple Sclerosis.” Lancet Neurology 3, no. 2: 104–110. 14747002 10.1016/s1474-4422(03)00663-x

[ejn70067-bib-0077] Dziedzic, A. , and M. Bijak . 2019. “Interactions Between Platelets and Leukocytes in Pathogenesis of Multiple Sclerosis.” Advances in Clinical and Experimental Medicine 28, no. 2: 277–285. 30411550 10.17219/acem/83588

[ejn70067-bib-0078] Dziedzic, A. , J. Saluk‐Bijak , E. Miller , and M. Bijak . 2020. “Metformin as a Potential Agent in the Treatment of Multiple Sclerosis.” International Journal of Molecular Sciences 21, no. 17: 5957. 32825027 10.3390/ijms21175957 PMC7503488

[ejn70067-bib-0079] El‐Saber Batiha, G. , A. I. Al‐Gareeb , H. M. Saad , and H. M. Al‐Kuraishy . 2022. “COVID‐19 and Corticosteroids: A Narrative Review.” Inflammopharmacology 30, no. 4: 1189–1205. 35562628 10.1007/s10787-022-00987-z PMC9106274

[ejn70067-bib-0080] Elsayed, N. S. , P. Aston , V. R. Bayanagari , and S. K. Shukla . 2022. “The Gut Microbiome Molecular Mimicry Piece in the Multiple Sclerosis Puzzle.” Frontiers in Immunology 13: 972160. 36045671 10.3389/fimmu.2022.972160 PMC9420973

[ejn70067-bib-0081] Fang, M. , L. Lu , J. Lou , et al. 2025. “FGF21 Alleviates Hypoxic‐Ischemic White Matter Injury in Neonatal Mice by Mediating Inflammation and Oxidative Stress Through PPAR‐γ Signaling Pathway.” Molecular Neurobiology 62, no. 4: 4743–4768. 39485628 10.1007/s12035-024-04549-y

[ejn70067-bib-0082] Flory, J. , and K. Lipska . 2019. “Metformin in 2019.” Journal of the American Medical Association 321, no. 19: 1926–1927. 31009043 10.1001/jama.2019.3805 PMC7552083

[ejn70067-bib-0083] Foretz, M. , B. Guigas , and B. Viollet . 2019. “Understanding the Glucoregulatory Mechanisms of Metformin in Type 2 Diabetes Mellitus.” Nature Reviews Endocrinology 15, no. 10: 569–589. 10.1038/s41574-019-0242-2 31439934

[ejn70067-bib-0084] Fortin, D. , E. Rom , H. Sun , A. Yayon , and R. Bansal . 2005. “Distinct Fibroblast Growth Factor (FGF)/FGF Receptor Signaling Pairs Initiate Diverse Cellular Responses in the Oligodendrocyte Lineage.” Journal of Neuroscience 25, no. 32: 7470–7479. 16093398 10.1523/JNEUROSCI.2120-05.2005 PMC6725305

[ejn70067-bib-0085] Gardón, D. P. , M. Cervantes‐Llanos , B. P. Matamoros , et al. 2022. “Positive Effects of Phycocyanobilin on Gene Expression in Glutamate‐Induced Excitotoxicity in SH‐SY5Y Cells and Animal Models of Multiple Sclerosis and Cerebral Ischemia.” Heliyon 8, no. 6: e09769. 35800718 10.1016/j.heliyon.2022.e09769 PMC9253351

[ejn70067-bib-0086] Gerdes, L. A. , C. Janoschka , M. Eveslage , et al. 2020. “Immune Signatures of Prodromal Multiple Sclerosis in Monozygotic Twins.” National Academy of Sciences of the United States of America 117, no. 35: 21546–21556. 10.1073/pnas.2003339117 PMC7474627 32817525

[ejn70067-bib-0087] Grant, P. 2003. “Beneficial Effects of Metformin on Haemostasis and Vascular Function in Man.” Diabetes & Metabolism 29, no. 4: 6S44–6S52. 14502100 10.1016/s1262-3636(03)72787-6

[ejn70067-bib-0088] Granziera, C. , J. Wuerfel , F. Barkhof , et al. 2021. “Quantitative Magnetic Resonance Imaging Towards Clinical Application in Multiple Sclerosis.” Brain 144, no. 5: 1296–1311. 33970206 10.1093/brain/awab029 PMC8219362

[ejn70067-bib-0090] Harroud, A. , D. Manousaki , G. Butler‐Laporte , et al. 2021. “The Relative Contributions of Obesity, Vitamin D, Leptin, and Adiponectin to Multiple Sclerosis Risk: A Mendelian Randomization Mediation Analysis.” Multiple Sclerosis Journal 27, no. 13: 1994–2000. 33605807 10.1177/1352458521995484

[ejn70067-bib-0091] Hasan Khudhair, D. , A. I. Al‐Gareeb , H. M. Al‐Kuraishy , et al. 2022. “Combination of Vitamin C and Curcumin Safeguards Against Methotrexate‐Induced Acute Liver Injury in Mice by Synergistic Antioxidant Effects.” Frontiers in Medicine 9: 866343. 35492324 10.3389/fmed.2022.866343 PMC9047671

[ejn70067-bib-0092] Hassan, F. I. , T. Didari , M. Baeeri , et al. 2020. “Metformin Attenuates Brain Injury by Inhibiting Inflammation and Regulating Tight Junction Proteins in Septic Rats.” Cell Journal (Yakhteh) 22, no. Suppl 1: 29. 10.22074/cellj.2020.7046 PMC7481907 32779431

[ejn70067-bib-0093] Hauser, S. L. , and B. A. Cree . 2020. “Treatment of Multiple Sclerosis: A Review.” American Journal of Medicine 133, no. 12: e2. 10.1016/j.amjmed.2020.05.049 PMC7704606 32682869

[ejn70067-bib-0094] He, L. 2020. “Metformin and Systemic Metabolism.” Trends in Pharmacological Sciences 41, no. 11: 868–881. 32994049 10.1016/j.tips.2020.09.001 PMC7572679

[ejn70067-bib-0095] Hedström, A. K. , O. Hössjer , J. Hillert , et al. 2020. “The Influence of Human Leukocyte Antigen‐DRB1* 15: 01 and Its Interaction With Smoking in MS Development Is Dependent on DQA1* 01: 01 Status.” Multiple Sclerosis Journal 26, no. 13: 1638–1646. 31573825 10.1177/1352458519877685

[ejn70067-bib-0096] Herath, P. M. , N. Cherbuin , R. Eramudugolla , and K. J. Anstey . 2016. “The Effect of Diabetes Medication on Cognitive Function: Evidence From the PATH Through Life Study.” BioMed Research International 2016, no. 1: 7208429. 27195294 10.1155/2016/7208429 PMC4853928

[ejn70067-bib-0097] Hussien, N. R. , M. S. Al‐Naimi , H. A. Rasheed , H. M. Al‐Kuraishy , and A. I. Al‐Gareeb . 2018. “Sulfonylurea and Neuroprotection: The Bright Side of the Moon.” Journal of Advanced Pharmaceutical Technology & Research 9, no. 4: 120–123. 30637228 10.4103/japtr.JAPTR_317_18 PMC6302683

[ejn70067-bib-0098] Imfeld, P. , M. Bodmer , S. S. Jick , and C. R. Meier . 2012. “Metformin, Other Antidiabetic Drugs, and Risk of Alzheimer's Disease: A Population‐Based Case–Control Study.” Journal of the American Geriatrics Society 60, no. 5: 916–921. 22458300 10.1111/j.1532-5415.2012.03916.x

[ejn70067-bib-0099] Jackson, T. C. , K. Janesko‐Feldman , S. W. Carlson , S. E. Kotermanski , and P. M. Kochanek . 2019. “Robust RBM3 and β‐Klotho Expression in Developing Neurons in the Human Brain.” Journal of Cerebral Blood Flow and Metabolism 39, no. 12: 2355–2367. 31566073 10.1177/0271678X19878889 PMC6890998

[ejn70067-bib-0100] James, R. E. , R. Schalks , E. Browne , et al. 2020. “Persistent Elevation of Intrathecal Pro‐Inflammatory Cytokines Leads to Multiple Sclerosis‐Like Cortical Demyelination and Neurodegeneration.” Acta Neuropathologica Communications 8, no. 1: 66. 10.1186/s40478-020-00938-1. 32398070 PMC7218553

[ejn70067-bib-0101] Kaikaryte, K. , G. Gedvilaite , R. Balnyte , I. Uloziene , and R. Liutkeviciene . 2023. “Role of SIRT1 Gene Polymorphisms and Serum Levels in Patients With Multiple Sclerosis.” Diagnostics 13, no. 20: 3287. 37892107 10.3390/diagnostics13203287 PMC10606525

[ejn70067-bib-0102] Kang, H. , R. Khang , S. Ham , et al. 2017. “Activation of the ATF2/CREB‐PGC‐1α Pathway by Metformin Leads to Dopaminergic Neuroprotection.” Oncotarget 8, no. 30: 48603. 28611284 10.18632/oncotarget.18122 PMC5564711

[ejn70067-bib-0103] Kar, E. , Ö. Alataş , V. Şahıntürk , and S. Öz . 2022. “Effects of Metformin on Lipopolysaccharide Induced Inflammation by Activating Fibroblast Growth Factor 21.” Biotechnic & Histochemistry 97, no. 1: 44–52. 33663305 10.1080/10520295.2021.1894353

[ejn70067-bib-0104] Karami, M. , F. Mehrabi , A. Allameh , M. P. Kakhki , M. Amiri , and M. S. E. Aleagha . 2017. “Klotho Gene Expression Decreases in Peripheral Blood Mononuclear Cells (PBMCs) of Patients With Relapsing‐Remitting Multiple Sclerosis.” Journal of the Neurological Sciences 381: 305–307. 28991703 10.1016/j.jns.2017.09.012

[ejn70067-bib-0105] Katsu‐Jiménez, Y. , and A. Giménez‐Cassina . 2019. “Fibroblast Growth Factor‐21 Promotes Ketone Body Utilization in Neurons Through Activation of AMP‐Dependent Kinase.” Molecular and Cellular Neurosciences 101: 103415. 31676432 10.1016/j.mcn.2019.103415

[ejn70067-bib-0106] Khatir, A. A. , S. M. M. Hojjati , A. A. Ahangar , H. Naghshineh , and P. Saadat . 2020. “Multiple Sclerosis and Its Pathophysiology: A Narrative Review.” Tabari Biomedical Student Research Journal 2, no. 2: 8–15.

[ejn70067-bib-0107] Kliewer, S. A. , and D. J. Mangelsdorf . 2010. “Fibroblast Growth Factor 21: From Pharmacology to Physiology.” American Journal of Clinical Nutrition 91, no. 1: 254S–257S. 19906798 10.3945/ajcn.2009.28449B PMC2793111

[ejn70067-bib-0108] Koudriavtseva, T. , and C. Mainero . 2016. “Neuroinflammation, Neurodegeneration and Regeneration in Multiple Sclerosis: Intercorrelated Manifestations of the Immune Response.” Neural Regeneration Research 11, no. 11: 1727–1730. 28123401 10.4103/1673-5374.194804 PMC5204213

[ejn70067-bib-0109] Krysiak, R. , A. Gdula‐Dymek , and B. Okopień . 2013. “Effect of Metformin on Selected Parameters of Hemostasis in Fenofibrate‐Treated Patients With Impaired Glucose Tolerance.” Pharmacological Reports 65: 208–213. 10.1016/S1734-1140(13)70980-0. 23563040

[ejn70067-bib-0110] Krysiak, R. , and B. Okopien . 2012. “Haemostatic Effects of Metformin in Simvastatin‐Treated Volunteers With Impaired Fasting Glucose.” Basic & Clinical Pharmacology & Toxicology 111, no. 6: 380–384. 22716204 10.1111/j.1742-7843.2012.00913.x

[ejn70067-bib-0111] Kumar, D. R. , F. Aslinia , S. H. Yale , and J. J. Mazza . 2011. “Jean‐Martin Charcot: The Father of Neurology.” Clinical Medicine & Research 9, no. 1: 46–49. 20739583 10.3121/cmr.2009.883 PMC3064755

[ejn70067-bib-0112] Kuroda, M. , R. Muramatsu , N. Maedera , et al. 2017. “Peripherally Derived FGF21 Promotes Remyelination in the Central Nervous System.” Journal of Clinical Investigation 127, no. 9: 3496–3509. 28825598 10.1172/JCI94337 PMC5669554

[ejn70067-bib-0113] Kurosu, H. , M. Choi , Y. Ogawa , et al. 2007. “Tissue‐Specific Expression of βKlotho and Fibroblast Growth Factor (FGF) Receptor Isoforms Determines Metabolic Activity of FGF19 and FGF21.” Journal of Biological Chemistry 282, no. 37: 26687–26695. 10.1074/jbc.M704165200. 17623664 PMC2496965

[ejn70067-bib-0114] LaMoia, T. E. , and G. I. Shulman . 2021. “Cellular and Molecular Mechanisms of Metformin Action.” Endocrine Reviews 42, no. 1: 77–96. 32897388 10.1210/endrev/bnaa023 PMC7846086

[ejn70067-bib-0115] Lan, M. , X. Tang , J. Zhang , and Z. Yao . 2017. “Insights in Pathogenesis of Multiple Sclerosis: Nitric Oxide May Induce Mitochondrial Dysfunction of Oligodendrocytes.” Reviews in the Neurosciences 29, no. 1: 39–53. 10.1515/revneuro-2017-0033 28822986

[ejn70067-bib-0116] Lane, J. , H. S. Ng , C. Poyser , R. M. Lucas , and H. Tremlett . 2022. “Multiple Sclerosis Incidence: A Systematic Review of Change Over Time by Geographical Region.” Multiple Sclerosis and Related Disorders 63: 103932. 35667315 10.1016/j.msard.2022.103932

[ejn70067-bib-0117] Largani, S. H. H. , M. Borhani‐Haghighi , P. Pasbakhsh , et al. 2019. “Oligoprotective Effect of Metformin Through the AMPK‐Dependent on Restoration of Mitochondrial Hemostasis in the Cuprizone‐Induced Multiple Sclerosis Model.” Journal of Molecular Histology 50: 263–271. 31016544 10.1007/s10735-019-09824-0

[ejn70067-bib-0118] Larson, K. R. , A. T. Chaffin , M. L. Goodson , Y. Fang , and K. K. Ryan . 2019. “Fibroblast Growth Factor‐21 Controls Dietary Protein Intake in Male Mice.” Endocrinology 160, no. 5: 1069–1080. 30802283 10.1210/en.2018-01056 PMC6469953

[ejn70067-bib-0119] Lassmann, H. , W. Brück , and C. Lucchinetti . 2001. “Heterogeneity of Multiple Sclerosis Pathogenesis: Implications for Diagnosis and Therapy.” Trends in Molecular Medicine 7, no. 3: 115–121. 11286782 10.1016/s1471-4914(00)01909-2

[ejn70067-bib-0120] Lee, J. Y. , Y. Kang , J. Y. Jeon , et al. 2024. “Imeglimin Attenuates NLRP3 Inflammasome Activation by Restoring Mitochondrial Functions in Macrophages.” Journal of Pharmacological Sciences 155, no. 2: 35–43. 38677784 10.1016/j.jphs.2024.03.004

[ejn70067-bib-0121] Lee, S.‐Y. , S.‐J. Moon , E.‐K. Kim , et al. 2017. “Metformin Suppresses Systemic Autoimmunity in Roquinsan/San Mice Through Inhibiting B Cell Differentiation Into Plasma Cells via Regulation of AMPK/mTOR/STAT3.” Journal of Immunology 198, no. 7: 2661–2670. 10.4049/jimmunol.1403088. PMC5357783 28242651

[ejn70067-bib-0122] Li, J. , J. Deng , W. Sheng , and Z. Zuo . 2012. “Metformin Attenuates Alzheimer's Disease‐Like Neuropathology in Obese, Leptin‐Resistant Mice.” Pharmacology Biochemistry and Behavior 101, no. 4: 564–574. 22425595 10.1016/j.pbb.2012.03.002 PMC3327803

[ejn70067-bib-0123] Li, K. , L. Li , M. Yang , H. Liu , G. Boden , and G. Yang . 2012. “The Effects of Fibroblast Growth Factor‐21 Knockdown and Over‐Expression on Its Signaling Pathway and Glucose–Lipid Metabolism In Vitro.” Molecular and Cellular Endocrinology 348, no. 1: 21–26. 21801806 10.1016/j.mce.2011.07.026

[ejn70067-bib-0124] Li, S. , H. Jia , Z. Liu , et al. 2022. “Fibroblast Growth Factor‐21 as a Novel Metabolic Factor for Regulating Thrombotic Homeostasis.” Scientific Reports 12, no. 1: 400. 35013379 10.1038/s41598-021-00906-2 PMC8748457

[ejn70067-bib-0125] Li, Y. , K. Song , H. Zhang , et al. 2020. “Anti‐Inflammatory and Immunomodulatory Effects of Baicalin in Cerebrovascular and Neurological Disorders.” Brain Research Bulletin 164: 314–324. 32858128 10.1016/j.brainresbull.2020.08.016

[ejn70067-bib-0126] Li, Y. , K. Wong , A. Giles , et al. 2014. “Hepatic SIRT1 Attenuates Hepatic Steatosis and Controls Energy Balance in Mice by Inducing Fibroblast Growth Factor 21.” Gastroenterology 146, no. 2: 539–549. 24184811 10.1053/j.gastro.2013.10.059 PMC4228483

[ejn70067-bib-0127] Linares, G. R. , Y. Leng , D. Maric , and D. M. Chuang . 2020. “Overexpression of Fibroblast Growth Factor‐21 (FGF‐21) Protects Mesenchymal Stem Cells Against Caspase‐Dependent Apoptosis Induced by Oxidative Stress and Inflammation.” Cell Biology International 44, no. 10: 2163–2169. 32557962 10.1002/cbin.11409 PMC10848314

[ejn70067-bib-0128] Liu, G.‐Z. , L.‐B. Fang , P. Hjelmström , and X.‐G. Gao . 2007. “Increased CD8+ Central Memory T Cells in Patients With Multiple Sclerosis.” Multiple Sclerosis Journal 13, no. 2: 149–155. 17439879 10.1177/1352458506069246

[ejn70067-bib-0129] Lovrecic, L. , S. Ristić , N. Starcević‐Cizmarević , et al. 2008. “PAI and TPA Gene Polymorphisms in Multiple Sclerosis.” Multiple Sclerosis 14: 243–247. 10.1177/1352458507082603. 17986506

[ejn70067-bib-0130] Luchsinger, J. A. , T. Perez , H. Chang , et al. 2016. “Metformin in Amnestic Mild Cognitive Impairment: Results of a Pilot Randomized Placebo Controlled Clinical Trial.” Journal of Alzheimer's Disease 51, no. 2: 501–514. 10.3233/JAD-150493 PMC5079271 26890736

[ejn70067-bib-0131] Maglinger, B. , J. A. Frank , C. J. McLouth , et al. 2021. “Proteomic Changes in Intracranial Blood During Human Ischemic Stroke.” Journal of NeuroInterventional Surgery 13, no. 4: 395–399. 10.1136/neurintsurg-2020-016118. 32641418 PMC7982920

[ejn70067-bib-0132] Maida, A. , B. Lamont , X. Cao , and D. Drucker . 2011. “Metformin Regulates the Incretin Receptor Axis via a Pathway Dependent on Peroxisome Proliferator‐Activated Receptor‐α in Mice.” Diabetologia 54: 339–349. 20972533 10.1007/s00125-010-1937-z

[ejn70067-bib-0133] Majeed, Y. , R. Upadhyay , A. Lakshmanan , C. Triggle , and H. Ding , eds. 2016. Down‐Regulation of Erk and Sirt1 Signaling May Lead to Reduced Fgf‐21 Sensitivity in a Mouse Model of Diabetes. Qatar Foundation Annual Research Conference Proceedings. HBKU Press Qatar.

[ejn70067-bib-0134] Mäkelä, J. , T. V. Tselykh , F. Maiorana , et al. 2014. “Fibroblast Growth Factor‐21 Enhances Mitochondrial Functions and Increases the Activity of PGC‐1α in Human Dopaminergic Neurons via Sirtuin‐1.” Springerplus 3: 1–12. 25932355 10.1186/2193-1801-3-2 PMC4409609

[ejn70067-bib-0135] Makhani, N. , and H. Tremlett . 2021. “The Multiple Sclerosis Prodrome.” Nature Reviews Neurology 17, no. 8: 515–521. 34155379 10.1038/s41582-021-00519-3 PMC8324569

[ejn70067-bib-0136] Markowicz‐Piasecka, M. , K. M. Huttunen , A. Sadkowska , and J. Sikora . 2019. “Pleiotropic Activity of Metformin and Its Sulfonamide Derivatives on Vascular and Platelet Haemostasis.” Molecules 25, no. 1: 125. 31905674 10.3390/molecules25010125 PMC6982810

[ejn70067-bib-0137] Marrie, R. A. 2004. “Environmental Risk Factors in Multiple Sclerosis Aetiology.” Lancet Neurology 3, no. 12: 709–718. 15556803 10.1016/S1474-4422(04)00933-0

[ejn70067-bib-0138] Martin, R. , M. Sospedra , T. Eiermann , and T. Olsson . 2021. “Multiple Sclerosis: Doubling Down on MHC.” Trends in Genetics 37, no. 9: 784–797. 34006391 10.1016/j.tig.2021.04.012

[ejn70067-bib-0139] Martino, G. , L. Adorini , P. Rieckmann , et al. 2002. “Inflammation in Multiple Sclerosis: The Good, the Bad, and the Complex.” Lancet Neurology 1, no. 8: 499–509. 12849335 10.1016/s1474-4422(02)00223-5

[ejn70067-bib-0140] Mayo, C. D. , K. Miksche , K. Attwell‐Pope , and J. R. Gawryluk . 2019. “The Relationship Between Physical Activity and Symptoms of Fatigue, Mood, and Perceived Cognitive Impairment in Adults With Multiple Sclerosis.” Journal of Clinical and Experimental Neuropsychology 41, no. 7: 715–722. 31096850 10.1080/13803395.2019.1614535

[ejn70067-bib-0141] McGinley, M. P. , C. H. Goldschmidt , and A. D. Rae‐Grant . 2021. “Diagnosis and Treatment of Multiple Sclerosis: A Review.” Journal of the American Medical Association 325, no. 8: 765–779. 33620411 10.1001/jama.2020.26858

[ejn70067-bib-0142] Md, C. 2010. “Fibroblast Growth Factor 21 Regulates Energy Metabolism by Activating the AMPK‐SIRT1‐PGC‐1α Pathway.” Proc Natl Acad Sci USA 107: 12553–12558. 20616029 10.1073/pnas.1006962107 PMC2906565

[ejn70067-bib-0143] Mehrabi, S. , N. Sanadgol , M. Barati , et al. 2018. “Evaluation of Metformin Effects in the Chronic Phase of Spontaneous Seizures in Pilocarpine Model of Temporal Lobe Epilepsy.” Metabolic Brain Disease 33: 107–114. 29080083 10.1007/s11011-017-0132-z

[ejn70067-bib-0144] Miclea, A. , M. Bagnoud , A. Chan , and R. Hoepner . 2020. “A Brief Review of the Effects of Vitamin D on Multiple Sclerosis.” Frontiers in Immunology 11: 781. 32435244 10.3389/fimmu.2020.00781 PMC7218089

[ejn70067-bib-0145] Mima, Y. , T. Kuwashiro , M. Yasaka , et al. 2016. “Impact of Metformin on the Severity and Outcomes of Acute Ischemic Stroke in Patients With Type 2 Diabetes Mellitus.” Journal of Stroke and Cerebrovascular Diseases 25, no. 2: 436–446. 26725260 10.1016/j.jstrokecerebrovasdis.2015.10.016

[ejn70067-bib-0146] Min, X. , J. Weiszmann , S. Johnstone , et al. 2018. “Agonistic β‐Klotho Antibody Mimics Fibroblast Growth Factor 21 (FGF21) Functions.” Journal of Biological Chemistry 293, no. 38: 14678–14688. 30068552 10.1074/jbc.RA118.004343 PMC6153294

[ejn70067-bib-0147] Mohammadhosayni, M. , A. Khosrojerdi , K. Lorian , et al. 2020. “Matrix Metalloproteinases (MMPs) Family Gene Polymorphisms and the Risk of Multiple Sclerosis: Systematic Review and Meta‐Analysis.” BMC Neurology 20, no. 1: 218. 10.1186/s12883-020-01804-2. 32471473 PMC7257507

[ejn70067-bib-0148] Moore, E. M. , A. G. Mander , D. Ames , et al. 2013. “Increased Risk of Cognitive Impairment in Patients With Diabetes Is Associated With Metformin.” Diabetes Care 36, no. 10: 2981–2987. 10.2337/dc13-0229. 24009301 PMC3781568

[ejn70067-bib-0149] Mostafa‐Hedeab, G. , H. M. Al‐Kuraishy , A. I. Al‐Gareeb , P. Jeandet , H. M. Saad , and G. E. Batiha . 2022. “A Raising Dawn of Pentoxifylline in Management of Inflammatory Disorders in Covid‐19.” Inflammopharmacology 30, no. 3: 799–809. 35486310 10.1007/s10787-022-00993-1 PMC9051499

[ejn70067-bib-0150] Moubarak, M. , K. I. Kasozi , H. F. Hetta , et al. 2021. “The Rise of SARS‐CoV‐2 Variants and the Role of Convalescent Plasma Therapy for Management of Infections.” Life 11, no. 8: 734. 34440478 10.3390/life11080734 PMC8399171

[ejn70067-bib-0151] Nath, N. , M. Khan , M. K. Paintlia , M. N. Hoda , and S. Giri . 2009. “Metformin Attenuated the Autoimmune Disease of the Central Nervous System in Animal Models of Multiple Sclerosis.” Journal of Immunology 182, no. 12: 8005–8014. 10.4049/jimmunol.0803563 PMC2965405 19494326

[ejn70067-bib-0152] Negrotto, L. , M. F. Farez , and J. Correale . 2016. “Immunologic Effects of Metformin and Pioglitazone Treatment on Metabolic Syndrome and Multiple Sclerosis.” JAMA Neurology 73, no. 5: 520–528. 26953870 10.1001/jamaneurol.2015.4807

[ejn70067-bib-0153] Neumann, B. , R. Baror , C. Zhao , et al. 2019. “Metformin Restores CNS Remyelination Capacity by Rejuvenating Aged Stem Cells.” Cell Stem Cell 25, no. 4: 473–485. 31585093 10.1016/j.stem.2019.08.015 PMC6863391

[ejn70067-bib-0154] Nijland, P. G. , M. E. Witte , B. van het Hof , et al. 2014. “Astroglial PGC‐1alpha Increases Mitochondrial Antioxidant Capacity and Suppresses Inflammation: Implications for Multiple Sclerosis.” Acta Neuropathologica Communications 2: 1–3. 25492529 10.1186/s40478-014-0170-2 PMC4268800

[ejn70067-bib-0155] Nygaard, E. B. , C. L. Møller , P. Kievit , K. L. Grove , and B. Andersen . 2014. “Increased Fibroblast Growth Factor 21 Expression in High‐Fat Diet‐Sensitive Non‐Human Primates (*Macaca mulatta*).” International Journal of Obesity 38, no. 2: 183–191. 23736354 10.1038/ijo.2013.79 PMC4376022

[ejn70067-bib-0156] Nygaard, E. B. , S. G. Vienberg , C. Ørskov , H. S. Hansen , and B. Andersen . 2012. “Metformin Stimulates FGF21 Expression in Primary Hepatocytes.” Journal Diabetes Research 2012, no. 1: 465282. 10.1155/2012/465282 PMC3478742 23118742

[ejn70067-bib-0157] Olcum, M. , B. Tastan , C. Kiser , S. Genc , and K. Genc . 2020. “Microglial NLRP3 Inflammasome Activation in Multiple Sclerosis.” Advances in Protein Chemistry and Structural Biology 119: 247–308. 31997770 10.1016/bs.apcsb.2019.08.007

[ejn70067-bib-0158] Onohuean, H. , H. M. Al‐Kuraishy , A. I. Al‐Gareeb , S. Qusti , E. M. Alshammari , and G. E. Batiha . 2021. “Covid‐19 and Development of Heart Failure: Mystery and Truth.” Naunyn‐Schmiedeberg's Archives of Pharmacology 394, no. 10: 2013–2021. 34480616 10.1007/s00210-021-02147-6 PMC8417660

[ejn70067-bib-0159] Ortiz, G. G. , F. P. Pacheco‐Moisés , M. Á. Macías‐Islas , et al. 2014. “Role of the Blood–Brain Barrier in Multiple Sclerosis.” Archives of Medical Research 45, no. 8: 687–697. 25431839 10.1016/j.arcmed.2014.11.013

[ejn70067-bib-0160] Ould‐Brahim, F. , S. N. Sarma , C. Syal , et al. 2018. “Metformin Preconditioning of Human Induced Pluripotent Stem Cell‐Derived Neural Stem Cells Promotes Their Engraftment and Improves Post‐Stroke Regeneration and Recovery.” Stem Cells and Development 27: 1085–1096. 10.1089/scd.2018.0055. 29893190

[ejn70067-bib-0161] Paintlia, A. S. , M. K. Paintlia , S. Mohan , A. K. Singh , and I. Singh . 2013. “AMP‐Activated Protein Kinase Signaling Protects Oligodendrocytes That Restore Central Nervous System Functions in an Experimental Autoimmune Encephalomyelitis Model.” American Journal of Pathology 183, no. 2: 526–541. 23759513 10.1016/j.ajpath.2013.04.030 PMC3730772

[ejn70067-bib-0162] Paudel, Y. N. , E. Angelopoulou , C. Piperi , M. F. Shaikh , and I. Othman . 2020. “Emerging Neuroprotective Effect of Metformin in Parkinson's Disease: A Molecular Crosstalk.” Pharmacological Research 152: 104593. 31843673 10.1016/j.phrs.2019.104593

[ejn70067-bib-0163] Pérez‐Martí, A. , V. Sandoval , P. F. Marrero , D. Haro , and J. Relat . 2017. “Nutritional Regulation of Fibroblast Growth Factor 21: From Macronutrients to Bioactive Dietary Compounds.” Hormone Molecular Biology and Clinical Investigation 30, no. 1: 20160034. 10.1515/hmbci-2016-0034 27583468

[ejn70067-bib-0164] Pitt, D. , C. H. Lo , S. A. Gauthier , et al. 2022. “Toward Precision Phenotyping of Multiple Sclerosis.” Neurology: Neuroimmunology & Neuroinflammation 9, no. 6: e200025. 10.1212/NXI.0000000000200025 PMC9427000 36041861

[ejn70067-bib-0165] Ponath, G. , C. Park , and D. Pitt . 2018. “The Role of Astrocytes in Multiple Sclerosis.” Frontiers in Immunology 9: 217. 29515568 10.3389/fimmu.2018.00217 PMC5826071

[ejn70067-bib-0166] Rasheed, H. A. , H. M. Al‐Kuraishy , A. I. Al‐Gareeb , N. R. Hussien , and M. S. Al‐Nami . 2019. “Effects of Diabetic Pharmacotherapy on Prolactin Hormone in Patients With Type 2 Diabetes Mellitus: Bane or Boon.” Journal of Advanced Pharmaceutical Technology & Research 10, no. 4: 163–168. 31742116 10.4103/japtr.JAPTR_65_19 PMC6844004

[ejn70067-bib-0167] Ren, H. , Y. Shao , C. Wu , X. Ma , C. Lv , and Q. Wang . 2020. “Metformin Alleviates Oxidative Stress and Enhances Autophagy in Diabetic Kidney Disease via AMPK/SIRT1‐FoxO1 Pathway.” Molecular and Cellular Endocrinology 500: 110628. 31647955 10.1016/j.mce.2019.110628

[ejn70067-bib-0168] Restelli, L. M. , B. Oettinghaus , M. Halliday , et al. 2018. “Neuronal Mitochondrial Dysfunction Activates the Integrated Stress Response to Induce Fibroblast Growth Factor 21.” Cell Reports 24, no. 6: 1407–1414. 30089252 10.1016/j.celrep.2018.07.023 PMC6092266

[ejn70067-bib-0169] Reuss, B. , and O. von Bohlen und Halbach . 2003. “Fibroblast Growth Factors and Their Receptors in the Central Nervous System.” Cell and Tissue Research 313: 139–157. 12845521 10.1007/s00441-003-0756-7

[ejn70067-bib-0170] Rice, G. P. , H.‐P. Hartung , and P. A. Calabresi . 2005. “Anti‐α4 Integrin Therapy for Multiple Sclerosis: Mechanisms and Rationale.” Neurology 64, no. 8: 1336–1342. 15851719 10.1212/01.WNL.0000158329.30470.D0

[ejn70067-bib-0171] Rodgers, M. , B. Heineman , and J. Dushay . 2019. “Increased Fructose Consumption Has Sex‐Specific Effects on Fibroblast Growth Factor 21 Levels in Humans.” Obesity Science & Practice 5, no. 5: 503–510. 31687174 10.1002/osp4.360 PMC6819978

[ejn70067-bib-0172] Ruddy, R. M. , K. V. Adams , and C. M. Morshead . 2019. “Age‐ and Sex‐Dependent Effects of Metformin on Neural Precursor Cells and Cognitive Recovery in a Model of Neonatal Stroke.” Science Advances 5: eaax1912. 10.1126/sciadv.aax1912. 31535024 PMC6739114

[ejn70067-bib-0173] Sag, D. , D. Carling , R. D. Stout , and J. Suttles . 2008. “Adenosine 5′‐Monophosphate‐Activated Protein Kinase Promotes Macrophage Polarization to an Anti‐Inflammatory Functional Phenotype.” Journal of Immunology 181, no. 12: 8633–8641. 10.4049/jimmunol.181.12.8633 PMC2756051 19050283

[ejn70067-bib-0174] Sakakibara, R. 2019. “Neurogenic Lower Urinary Tract Dysfunction in Multiple Sclerosis, Neuromyelitis Optica, and Related Disorders.” Clinical Autonomic Research 29: 313–320. 30076494 10.1007/s10286-018-0551-x

[ejn70067-bib-0175] Sanadgol, N. , M. Barati , F. Houshmand , et al. 2020. “Metformin Accelerates Myelin Recovery and Ameliorates Behavioral Deficits in the Animal Model of Multiple Sclerosis via Adjustment of AMPK/Nrf2/mTOR Signaling and Maintenance of Endogenous Oligodendrogenesis During Brain Self‐Repairing Period.” Pharmacological Reports 72: 641–658. 32048246 10.1007/s43440-019-00019-8

[ejn70067-bib-0176] Sanz, P. , J. M. Serratosa , and M. P. Sánchez . 2021. “Beneficial Effects of Metformin on the Central Nervous System, With a Focus on Epilepsy and Lafora Disease.” International Journal of Molecular Sciences 22, no. 10: 5351. 34069559 10.3390/ijms22105351 PMC8160983

[ejn70067-bib-0177] Scazzone, C. , L. Agnello , B. L. Sasso , et al. 2019. “Klotho and Vitamin D in Multiple Sclerosis: An Italian Study.” Archives of Medical Science 16, no. 4: 842–847. 32542086 10.5114/aoms.2019.86969 PMC7286339

[ejn70067-bib-0178] Sedel, F. , D. Bernard , D. M. Mock , and A. Tourbah . 2016. “Targeting Demyelination and Virtual Hypoxia With High‐Dose Biotin as a Treatment for Progressive Multiple Sclerosis.” Neuropharmacology 110: 644–653. 26327679 10.1016/j.neuropharm.2015.08.028

[ejn70067-bib-0179] Serdyńska‐Szuster, M. , B. Banaszewska , R. Spaczyński , and L. Pawelczyk . 2011. “Effects of Metformin Therapy on Markers of Coagulation Disorders in Hyperinsulinemic Women With Polycystic Ovary Syndrome.” Ginekologia Polska 82, no. 4: 259–264. 21735693

[ejn70067-bib-0180] Sf, G. 2021. “The Relationship Between Neutrophil/Lymphocyte Ratio and Uric Acid Levels in Multiple Sclerosis Patients.” Bratislava Medical Journal/Bratislavské Lekárske Listy 122, no. 5: 357–361. 33848187 10.4149/BLL_2021_060

[ejn70067-bib-0181] Shahror, R. A. , G. R. Linares , Y. Wang , et al. 2020. “Transplantation of Mesenchymal Stem Cells Overexpressing Fibroblast Growth Factor 21 Facilitates Cognitive Recovery and Enhances Neurogenesis in a Mouse Model of Traumatic Brain Injury.” Journal of Neurotrauma 37, no. 1: 14–26. 31298621 10.1089/neu.2019.6422 PMC6921331

[ejn70067-bib-0182] Sharma, N. , A. Shandilya , N. Kumar , and S. Mehan . 2021. “Dysregulation of SIRT‐1 Signaling in Multiple Sclerosis and Neuroimmune Disorders: A Systematic Review of SIRTUIN Activators as Potential Immunomodulators and Their Influences on Other Dysfunctions.” Endocrine, Metabolic & Immune Disorders Drug Targets 21, no. 10: 1845–1868. 10.2174/1871530321666210309112234 33687904

[ejn70067-bib-0183] Sharma, S. , S. Nozohouri , B. Vaidya , and T. Abbruscato . 2021. “Repurposing Metformin to Treat Age‐Related Neurodegenerative Disorders and Ischemic Stroke.” Life Sciences 274: 119343. 33716063 10.1016/j.lfs.2021.119343 PMC8996678

[ejn70067-bib-0184] Singh, R. , S. C. Sarangi , S. Singh , and M. Tripathi . 2022. “A Review on Role of Metformin as a Potential Drug for Epilepsy Treatment and Modulation of Epileptogenesis.” Seizure 101: 253–261. 36116284 10.1016/j.seizure.2022.09.003

[ejn70067-bib-0185] Singhal, G. , F. M. Fisher , M. J. Chee , et al. 2016. “Fibroblast Growth Factor 21 (FGF21) Protects Against High Fat Diet Induced Inflammation and Islet Hyperplasia in Pancreas.” PLoS ONE 11, no. 2: e0148252. 10.1371/journal.pone.0148252. 26872145 PMC4752212

[ejn70067-bib-0186] So, W. Y. , and P. S. Leung . 2016. “Fibroblast Growth Factor 21 as an Emerging Therapeutic Target for Type 2 Diabetes Mellitus.” Medicinal Research Reviews 36, no. 4: 672–704. 27031294 10.1002/med.21390

[ejn70067-bib-0187] Standeven, K. F. , R. A. Ariëns , P. Whitaker , A. E. Ashcroft , J. W. Weisel , and P. J. Grant . 2002. “The Effect of Dimethylbiguanide on Thrombin Activity, FXIII Activation, Fibrin Polymerization, and Fibrin Clot Formation.” Diabetes 51, no. 1: 189–197. 11756340 10.2337/diabetes.51.1.189

[ejn70067-bib-0188] Storer, P. D. , J. Xu , J. Chavis , and P. D. Drew . 2005. “Peroxisome Proliferator‐Activated Receptor‐Gamma Agonists Inhibit the Activation of Microglia and Astrocytes: Implications for Multiple Sclerosis.” Journal of Neuroimmunology 161, no. 1–2: 113–122. 15748950 10.1016/j.jneuroim.2004.12.015

[ejn70067-bib-0189] Su, J. J. , M. Osoegawa , T. Matsuoka , et al. 2006. “Upregulation of Vascular Growth Factors in Multiple Sclerosis: Correlation With MRI Findings.” Journal of the Neurological Sciences 243, no. 1–2: 21–30. 16376944 10.1016/j.jns.2005.11.006

[ejn70067-bib-0190] Sun, H. , M. Sherrier , and H. Li . 2021. “Skeletal Muscle and Bone–Emerging Targets of Fibroblast Growth Factor‐21.” Frontiers in Physiology 12: 625287. 33762965 10.3389/fphys.2021.625287 PMC7982600

[ejn70067-bib-0191] Sun, Y. , Y. Wang , S.‐T. Chen , et al. 2020. “Modulation of the Astrocyte‐Neuron Lactate Shuttle System Contributes to Neuroprotective Action of Fibroblast Growth Factor 21.” Theranostics 10, no. 18: 8430–8445. 32724479 10.7150/thno.44370 PMC7381735

[ejn70067-bib-0192] Takeda, Y. , S.‐i. Fujita , T. Ikemoto , et al. 2015. “The Relationship of Fibroblast Growth Factors 21 and 23 and α‐Klotho With Platelet Activity Measured by Platelet Volume Indices.” Clinical Chemistry and Laboratory Medicine 53, no. 10: 1569–1574. 10.1515/cclm-2014-1251. 25781694

[ejn70067-bib-0193] Tan, B. K. , M. Hallschmid , R. Adya , W. Kern , H. Lehnert , and H. S. Randeva . 2011. “Fibroblast Growth Factor 21 (FGF21) in Human Cerebrospinal Fluid: Relationship With Plasma FGF21 and Body Adiposity.” Diabetes 60, no. 11: 2758–2762. 21926274 10.2337/db11-0672 PMC3198100

[ejn70067-bib-0194] Tarry‐Adkins, J. L. , S. E. Ozanne , and C. E. Aiken . 2021. “Impact of Metformin Treatment During Pregnancy on Maternal Outcomes: A Systematic Review/Meta‐Analysis.” Scientific Reports 11, no. 1: 9240. 33927270 10.1038/s41598-021-88650-5 PMC8085032

[ejn70067-bib-0195] Tegla, C. A. , P. Azimzadeh , M. Andrian‐Albescu , et al. 2014. “SIRT1 Is Decreased During Relapses in Patients With Multiple Sclerosis.” Experimental and Molecular Pathology 96, no. 2: 139–148. 24397908 10.1016/j.yexmp.2013.12.010

[ejn70067-bib-0196] Teoli, D , F. Rocha Cabrero , and S. Ghassemzadeh . 2021. “Lhermitte Sign.” StatPearls. Treasure Island, FL. StatPearls Publishing Copyright. 29630289

[ejn70067-bib-0197] Tintore, M. , A. Vidal‐Jordana , and J. Sastre‐Garriga . 2019. “Treatment of Multiple Sclerosis—Success From Bench to Bedside.” Nature Reviews Neurology 15, no. 1: 53–58. 30315270 10.1038/s41582-018-0082-z

[ejn70067-bib-0198] Trueck, C. , C. Hsin , O. Scherf‐Clavel , et al. 2019. “A Clinical Drug‐Drug Interaction Study Assessing a Novel Drug Transporter Phenotyping Cocktail With Adefovir, Sitagliptin, Metformin, Pitavastatin, and Digoxin.” Clinical Pharmacology and Therapeutics 106, no. 6: 1398–1407. 31247117 10.1002/cpt.1564

[ejn70067-bib-0199] Turkistani, A. , H. M. Al‐Kuraishy , A. I. Al‐Gareeb , et al. 2024. “Pharmacological Characterization of the Antidiabetic Drug Metformin in Atherosclerosis Inhibition: A Comprehensive Insight.” Immunity, Inflammation and Disease 12, no. 8: e1346. 39092773 10.1002/iid3.1346 PMC11295104

[ejn70067-bib-0200] Uebanso, T. , Y. Taketani , H. Yamamoto , et al. 2012. “Liver X Receptor Negatively Regulates Fibroblast Growth Factor 21 in the Fatty Liver Induced by Cholesterol‐Enriched Diet.” Journal of Nutritional Biochemistry 23, no. 7: 785–790. 21889884 10.1016/j.jnutbio.2011.03.023

[ejn70067-bib-0201] Vazifehkhah, S. , A. M. Khanizadeh , T. B. Mojarad , and F. Nikbakht . 2020. “The Possible Role of Progranulin on Anti‐Inflammatory Effects of Metformin in Temporal Lobe Epilepsy.” Journal of Chemical Neuroanatomy 109: 101849. 32679167 10.1016/j.jchemneu.2020.101849

[ejn70067-bib-0202] Vecchio, F. , F. Miraglia , C. Porcaro , et al. 2017. “Electroencephalography‐Derived Sensory and Motor Network Topology in Multiple Sclerosis Fatigue.” Neurorehabilitation and Neural Repair 31, no. 1: 56–64. 27370602 10.1177/1545968316656055

[ejn70067-bib-0203] Venna, V. R. , J. Li , M. D. Hammond , N. S. Mancini , and L. D. McCullough . 2014. “Chronic Metformin Treatment Improves Post‐Stroke Angiogenesis and Recovery After Experimental Stroke.” European Journal of Neuroscience 39, no. 12: 2129–2138. 24649970 10.1111/ejn.12556 PMC4061245

[ejn70067-bib-0204] Vespignani, M. 2020. “Integrative Approaches to Multiple Sclerosis.” Integrative Neurology 219–238.32471443

[ejn70067-bib-0205] Wachowicz, B. , A. Morel , E. D. Miller , and J. Saluk . 2016. “The Physiology of Blood Platelets and Changes of Their Biological Activities in Multiple Sclerosis.” Acta Neurobiologiae Experimentalis 76, no. 4: 269–281. 28094818 10.21307/ane-2017-026

[ejn70067-bib-0206] Wan, Y. 2013. “Bone Marrow Mesenchymal Stem Cells: Fat on and Blast Off by FGF21.” International Journal of Biochemistry & Cell Biology 45, no. 3: 546–549. 23270727 10.1016/j.biocel.2012.12.014 PMC3568182

[ejn70067-bib-0207] Wang, C. , C. Liu , K. Gao , et al. 2016. “Metformin Preconditioning Provide Neuroprotection Through Enhancement of Autophagy and Suppression of Inflammation and Apoptosis After Spinal Cord Injury.” Biochemical and Biophysical Research Communications 477, no. 4: 534–540. 27246734 10.1016/j.bbrc.2016.05.148

[ejn70067-bib-0208] Wang, H. , Z. Zheng , W. Han , et al. 2020. “Metformin Promotes Axon Regeneration After Spinal Cord Injury Through Inhibiting Oxidative Stress and Stabilizing Microtubule.” Oxidative Medicine and Cellular Longevity 2020, no. 1: 9741369. 31998447 10.1155/2020/9741369 PMC6969994

[ejn70067-bib-0209] Wang, X.‐M. , H. Xiao , L.‐L. Liu , D. Cheng , X.‐J. Li , and L.‐Y. Si . 2016. “FGF21 Represses Cerebrovascular Aging via Improving Mitochondrial Biogenesis and Inhibiting p53 Signaling Pathway in an AMPK‐Dependent Manner.” Experimental Cell Research 346, no. 2: 147–156. 27364911 10.1016/j.yexcr.2016.06.020

[ejn70067-bib-0210] Wijnen, J. , I. Van De Riet , W. Lijfering , and F. Van Der Meer . 2014. “Metformin use Decreases the Anticoagulant Effect of Phenprocoumon.” Journal of Thrombosis and Haemostasis 12, no. 6: 887–890. 24698366 10.1111/jth.12578

[ejn70067-bib-0211] Witte, M. E. , P. G. Nijland , J. A. Drexhage , et al. 2013. “Reduced Expression of PGC‐1α Partly Underlies Mitochondrial Changes and Correlates With Neuronal Loss in Multiple Sclerosis Cortex.” Acta Neuropathologica 125, no. 2: 231–243. 10.1007/s00401-012-1052-y. 23073717

[ejn70067-bib-0212] Woodbury, M. E. , and T. Ikezu . 2014. “Fibroblast Growth Factor‐2 Signaling in Neurogenesis and Neurodegeneration.” Journal of Neuroimmune Pharmacology 9: 92–101. 24057103 10.1007/s11481-013-9501-5 PMC4109802

[ejn70067-bib-0213] Xin, G. , Z. Wei , C. Ji , et al. 2016. “Metformin Uniquely Prevents Thrombosis by Inhibiting Platelet Activation and mtDNA Release.” Scientific Reports 6, no. 1: 36222. 27805009 10.1038/srep36222 PMC5090250

[ejn70067-bib-0214] Xu, J. , M. K. Racke , and P. D. Drew . 2007. “Peroxisome Proliferator‐Activated Receptor‐α Agonist Fenofibrate Regulates IL‐12 Family Cytokine Expression in the CNS: Relevance to Multiple Sclerosis.” Journal of Neurochemistry 103, no. 5: 1801–1810. 17727629 10.1111/j.1471-4159.2007.04875.x PMC2288776

[ejn70067-bib-0215] Xu, T. , X. Wu , X. Lu , et al. 2021. “Metformin Activated AMPK Signaling Contributes to the Alleviation of LPS‐Induced Inflammatory Responses in Bovine Mammary Epithelial Cells.” BMC Veterinary Research 17: 1–5. 33648513 10.1186/s12917-021-02797-x PMC7923493

[ejn70067-bib-0216] Yang, C. , W. Wang , P. Deng , C. Li , L. Zhao , and H. Gao . 2021. “Fibroblast Growth Factor 21 Modulates Microglial Polarization That Attenuates Neurodegeneration in Mice and Cellular Models of Parkinson's Disease.” Frontiers in Aging Neuroscience 13: 778527. 35002679 10.3389/fnagi.2021.778527 PMC8727910

[ejn70067-bib-0217] Yates, R. L. , M. M. Esiri , J. Palace , B. Jacobs , R. Perera , and G. C. DeLuca . 2017. “Fibrin (Ogen) and Neurodegeneration in the Progressive Multiple Sclerosis Cortex.” Annals of Neurology 82, no. 2: 259–270. 28719020 10.1002/ana.24997

[ejn70067-bib-0218] Yu, J. 2015. “Conjugated Linoleic Acid Induces Hepatic Expression of Fibroblast Growth Factor 21 Through PPAR‐Î±”. 10.1017/S0007114511003205 21767451

[ejn70067-bib-0219] Yu, Y. , F. Bai , W. Wang , et al. 2015. “Fibroblast Growth Factor 21 Protects Mouse Brain Against D‐Galactose Induced Aging via Suppression of Oxidative Stress Response and Advanced Glycation End Products Formation.” Pharmacology Biochemistry and Behavior 133: 122–131. 25871519 10.1016/j.pbb.2015.03.020

[ejn70067-bib-0220] Yu, Y. , J. He , S. Li , et al. 2016. “Fibroblast Growth Factor 21 (FGF21) Inhibits Macrophage‐Mediated Inflammation by Activating Nrf2 and Suppressing the NF‐κB Signaling Pathway.” International Immunopharmacology 38: 144–152. 27276443 10.1016/j.intimp.2016.05.026

[ejn70067-bib-0221] Zemgulyte, G. , D. Umbrasas , P. Cizas , et al. 2022. “Imeglimin Is Neuroprotective Against Ischemic Brain Injury in Rats—A Study Evaluating Neuroinflammation and Mitochondrial Functions.” Molecular Neurobiology 59, no. 5: 2977–2991. 35257284 10.1007/s12035-022-02765-y

[ejn70067-bib-0222] Zeydan, B. , and O. H. Kantarci . 2020. “Impact of Age on Multiple Sclerosis Disease Activity and Progression.” Current Neurology and Neuroscience Reports 20: 1–7. 32458308 10.1007/s11910-020-01046-2

[ejn70067-bib-0223] Zhang, M. , Y. Liu , Z.‐y. Xiong , Z.‐y. Deng , H.‐l. Song , and Z.‐M. An . 2013. “Changes of Plasma Fibroblast Growth Factor‐21 (FGF‐21) in Oral Glucose Tolerance Test and Effects of Metformin on FGF‐21 Levels in Type 2 Diabetes Mellitus.” Endokrynologia Polska 64, no. 3: 220–224. 23873427

[ejn70067-bib-0224] Zhang, Y. , S. Choksi , K. Chen , Y. Pobezinskaya , I. Linnoila , and Z.‐G. Liu . 2013. “ROS Play a Critical Role in the Differentiation of Alternatively Activated Macrophages and the Occurrence of Tumor‐Associated Macrophages.” Cell Research 23, no. 7: 898–914. 23752925 10.1038/cr.2013.75 PMC3698641

[ejn70067-bib-0225] Zhou, C. , R. Sun , S. Zhuang , et al. 2016. “Metformin Prevents Cerebellar Granule Neurons Against Glutamate‐Induced Neurotoxicity.” Brain Research Bulletin 121: 241–245. 26876755 10.1016/j.brainresbull.2016.02.009

[ejn70067-bib-0226] Zhou, Z. , W. Sun , Y. Liang , et al. 2012. “Fenofibrate Inhibited the Differentiation of T Helper 17 Cells In Vitro.” PPAR Research 2012, no. 1: 145654. 22792085 10.1155/2012/145654 PMC3388320

